# Social subordination induced by early life adversity rewires inhibitory control of the prefrontal cortex via enhanced *Npy1r* signaling

**DOI:** 10.1038/s41386-020-0727-7

**Published:** 2020-06-03

**Authors:** Lara O. Franco, Mário J. Carvalho, Jéssica Costa, Pedro A. Ferreira, Joana R. Guedes, Renato Sousa, Mohamed Edfawy, Catarina M. Seabra, Ana L. Cardoso, João Peça

**Affiliations:** 1 CNC—Center for Neuroscience and Cell Biology, University of Coimbra, Coimbra, Portugal; 2 Institute for Interdisciplinary Research, University of Coimbra, Coimbra, Portugal; 3 PhD Program in Experimental Biology and Biomedicine (PDBEB), University of Coimbra, Coimbra, Portugal; 4 MIT-Portugal Bioengineering Systems Doctoral Program, Coimbra, Portugal; 5 Department of Life Sciences, University of Coimbra, Coimbra, Portugal

**Keywords:** Social neuroscience, Risk factors, Stress and resilience

## Abstract

Social hierarchies are present in most mammalian species. In nature, hierarchies offer a tradeoff between reduction of in-group fighting between males, at the expense of an asymmetric sharing of resources. Early life experiences and stress are known to influence the rank an individual attains in adulthood, but the associated cellular and synaptic alterations are poorly understood. Using a maternal separation protocol, we show that care-deprived mice display a long-lasting submissive phenotype, increased social recognition, and enhanced explorative behavior. These alterations are consistent with an adaptation that favors exploration rather than confrontation within a group setting. At the neuronal level, these animals display dendritic atrophy and enhanced inhibitory synaptic inputs in medial prefrontal cortex (mPFC) neurons. To determine what could underlie this synaptic modification, we first assessed global gene expression changes via RNAseq, and next focused on a smaller subset of putatively altered synaptic receptors that could explain the changes in synaptic inhibition. Using different cohorts of maternally deprived mice, we validated a significant increase in the expression of *Npy1r*, a receptor known to play a role in maternal care, anxiety, foraging, and regulation of group behavior. Using electrophysiological recordings in adult mice while blocking NPY1R signaling, we determined that this receptor plays a key role in enhancing GABAergic currents in mice that experience maternal deprivation. Taken together, our work highlights the potential of regulating NPY1R in social anxiety disorders and the alterations induced in brain circuitry as a consequence of early life stress and adversity.

## Introduction

Individuals from gregarious species experience social stressors emanating from isolation, agonistic encounters, overcrowding, or from the relative rank in their social hierarchy [1–4]. As a physiological insult, social stressors produce salient responses along the animals’ lifespan [5], such that in primates, stress linked to the position an individual occupies within its hierarchy is the single best predictor of health [2]. In the mammalian brain, the medial prefrontal cortex (mPFC) has been implicated in the control of social hierarchy behaviors [6, 7] and is a central target for neuroendocrine signals [8, 9]. Persistent activation of the hypothalamic-pituitary-adrenal axis produces a remodeling in the mPFC that impacts decision-making [10]. This brain area is also critical in processing social and moral reasoning in humans [7, 11] and, in rodents, damaging the mPFC lowers social dominance and induces deficits in recognizing social rank [12, 13]. Recent studies have demonstrated that manipulation of synaptic strength in this area alone is sufficient to provide direct and bidirectional control over social rank [6, 14]. Together, these data indicate that mPFC circuitry is reciprocally involved in biological aspects pertaining to the control of social dominance and in the integration of stress response.

In mice, early life stress (ELS) induced by maternal separation leads to a lower rank in adulthood [15, 16]. However, the physiological alterations behind these changes in behavior have not been fully explored. Here, using behavior tests, RNAseq gene expression analysis, neuronal morphology studies, and electrophysiology recordings, we uncover a striking remodeling in prefrontal synaptic network function that occurs as a consequence of adverse rearing conditions induced by maternal separation. At the behavioral level, ELS male mice display social subordination, enhanced social recognition, and increased explorative behavior. From a large pool of gene expression changes induced in the mPFC by ELS, we pinpoint *Npy1r* as an element whose expression level is strongly and inversely correlated with social dominance. We also find evidence that NPY1R is involved in mediating alterations in synaptic inhibitory currents in the mPFC of ELS mice. More broadly, the ethological significance of these findings suggests that, in mice, adverse or disadvantageous rearing produces neuronal and gene expression changes that favor social behavioral adaptations that drive individuals toward egress (exploration) rather than fighting within the social hierarchy (exploitation).

## Materials and methods

Detailed methods for behavioral tests, electrophysiology recordings, neuronal labeling with AAV, and confocal imaging, preparation of RNA and DNA samples, RNAseq, methylation assays, qRT-PCR, immunofluorescence, and tissue dissection methodologies were performed as previously described [17–19] and are provided as Supplementary materials and methods.

### Animals

Mouse cages were maintained at a constant temperature (22 °C) and humidity (60%), under a 12 h light/dark cycle (lights on from 7 a.m. to 7 p.m.) in an individual cage ventilation system. After weaning, experimental animals were raised in groups of four mice per cage and allowed access to water and food *ad libitum*. Only male animals were used in this study. Tests were conducted from 9 am to 5 pm. Maintenance and handling of animals was performed according to the Animals Use and Care Guidelines issued by FELASA. All experiments with mice were carried out according to the protocols approved by ORBEA (Institutional Animal Welfare Body of the University of Coimbra), DGAV (Portuguese Regulatory Agency), and European Directives on Animal Welfare. All behavioral tests and quantifications were performed by trained experimentalists blinded to animal treatment. Behavioral tests were performed in age-matched 3–6-month-old animals.

### ELS induced by maternal separation and maternal unpredictable stress

The maternal separation and maternal unpredictable stress protocol was adapted from [20]. Briefly, after mating, pregnant females were individually housed and daily inspected for delivery (assigned as day 0). During the first 2 postnatal weeks, from postnatal day (PND) 2 to PND 14, daily maternal separation of 3 h was performed during the dark cycle by placing mothers and pups in two separate clean cages. Dams were allowed access to water and food *ad libitum*, and pups remained together during the separation period. Cages were placed side by side to allow visual and olfactory contact. Timing of separation and maternal stress was performed on a pseudo-random schedule. To perform unpredictable maternal stress, we used either a 20-min restraint in a plexiglas tube or of a 5-min forced swim in cold water (18 °C). Control litters were left undisturbed except for cage maintenance. All pups were weighted on PND 2, 7, 14, and 21. After weaning (PND 21), males from the same treatment group were reared in social groups of four mice per cage.

### Statistical analysis

Data are represented as mean values ± s.e.m. or as frequency distribution plots (as indicated in figure legend). Statistical analysis was performed using unpaired two-tailed Student’s *t* test, two-tailed Mann–Whitney test, one-way, or two-way ANOVA analysis followed by Bonferroni, Sidak’s or FDR post-hoc test (indicated where applicable), or one-sample Chi-square test. Sample normality was tested using D’Agostino–Pearson normality test. Analysis was performed using Prism (Graphpad) or MATLAB (Mathworks). Statistical significance was defined as ***p < 0.001, **p < 0.01, *p < 0.05.

## Results

### Early life adversity induces social subordination and increased explorative behavior

**Fig. 1:**
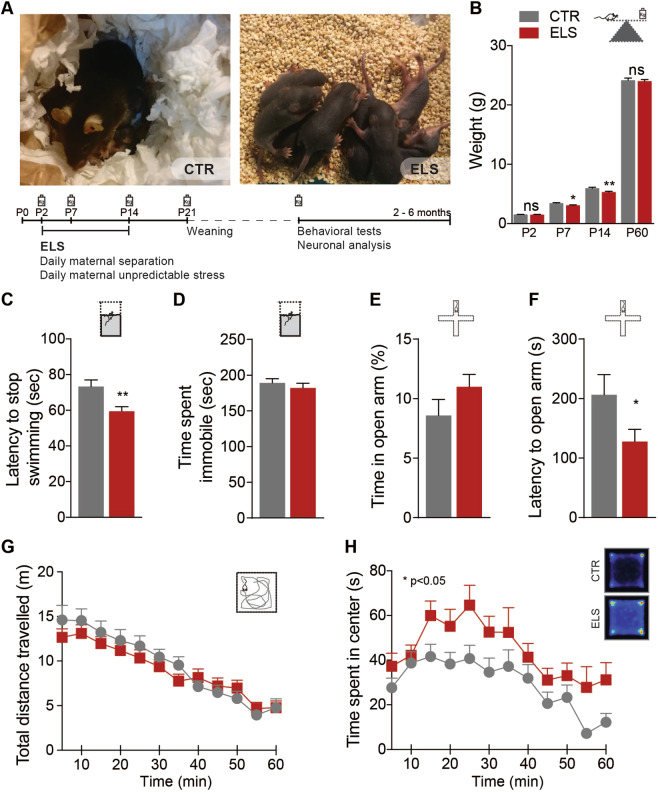
ELS mice show enhanced risk-taking and depressive-like behaviors. **a** Experimental design to induce and assess the effects of ELS on adult male mice (2–3 months old). **b** When compared with CTR, the weight of ELS animals is lower during the period of maternal separation but normalizes when animals reach P60. **c**, **d** ELS induces a decrease in latency to stop swimming in the forced swimming test, but no change in total time spent immobile. **e**, **f** Alterations in anxiety-like behaviors were assessed in the elevated plus maze including (**d**) percentage of time spent in the open arms and (**e**) latency to enter an open arm. ELS mice display normal motor but increased exploratory behavior in the open field, when measuring (**g**) total distance traveled and (**h**) time spent in the center portion of the arena, respectively. Statistical comparisons were performed using unpaired two-tailed *t*-test (for **b**–**f**) and two-way repeated measures ANOVA (for **g**, **h**); CTR *n* = 18–25, ELS *n* = 24–35. Statistical significance was set as *p < 0.05; **p < 0.01. Data are presented as means ± SEM.

To address the consequences of ELS, we subjected C57BL/6 pups to unpredictable maternal separation and maternal stress between PND 2 and PND 14 (Fig. 1a). ELS mice initially displayed lower body weight during the period of separation but recovered fully to similar levels as control animals (CTR) by 2 months of age (Fig. 1b). In our battery of behavioral tests (Fig. 1c–h) ELS male mice did not display gross motor behavior deficits (Fig. 1g). However, as previously reported [21], we found alterations in the forced swimming test, seen as a decrease in the latency to stop swimming (CTR: 73.32 ± 3.666 s, *n* = 25; ELS: 59.46 ± 2.588 s, *n* = 35; *p* = 0.0023) (Fig. 1c), however, there were no significant changes between groups in terms of time spent immobile (CTR: 189.0 ± 6.097 s, *n* = 25; ELS: 182.2 ± 6.478 s, *n* = 35; *p* > 0.05) (Fig. 1d). We observed low anxiety levels in ELS animals, as the latency to enter the open arms in the elevated plus maze was reduced (CTR: 206 ± 34.21 s, *n* = 24; ELS: 127.6 ± 20.7 s, *n* = 35; *p* = 0.0421) (Fig. 1f), and total time spent in the center of the open field was increased (two-way ANOVA CTR × ELS; *F*_(1, 40)_ = 5.719; *p* = 0.0216) (Fig. 1h). We then tested a separate cohort of animals in an exploration test designed to assess the latency to bite a food pellet placed in the center of a novel open field and again observed a risk-taking behavior by ELS animals (CTR: 284.1 ± 13.52 s, *n* = 7; ELS: 207.9 ± 26.89 s, *n* = 7; *p* = 0.0256) (Supplementary Fig. 1A). This difference was no longer present when the test was performed in a clean home cage (Supplementary Fig. 1B). Together, these results suggest that ELS induced an increase in explorative risk-taking behavior.

**Fig. 2:**
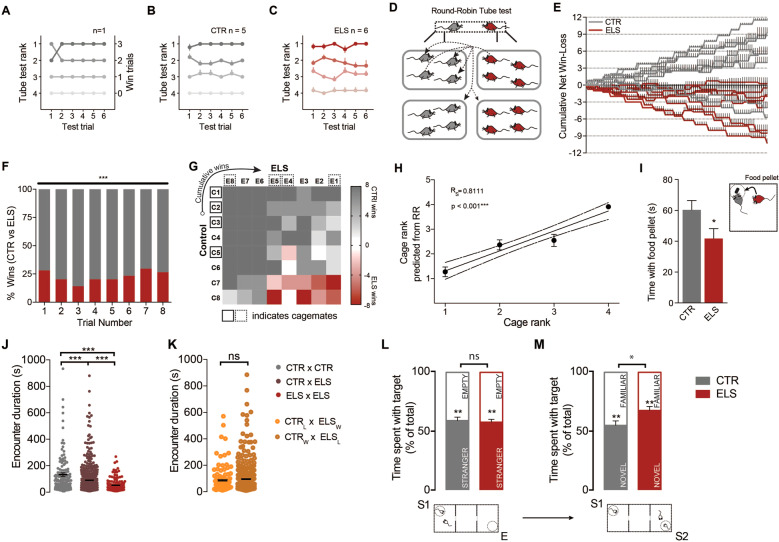
Social subordinate behavior and enhanced social recognition in ELS mice. **a** Example of an intra-cage hierarchy structure and social ranks for a single cage with four adult CTR cage-mates tested daily for 6 days in a tube test. Rank stability is observed in both (**b**) CTR and (**c**) ELS cages, with rare rank transitions; CTR *n* = 5 cages, ELS *n* = 6 cages. **d** Schematic representation of encounters performed by each animal in a round-robin test, where each animal only competes with mice from other cages in a total of 12 dyadic encounters per trial. **e** Meta-hierarchy between unfamiliar CTR and ELS adult male mice is represented by the cumulative performance of each animal across each encounter, with error bar representing deviation in encounter outcome across trials; CTR *n* = 8, ELS *n* = 8, across 8 RRTT trials. **f** When compared across time, social subordination in ELS mice presents a stable overall percentage of win–loss encounters when assessing CTR–ELS dyadic encounters; CTR *n* = 8, ELS *n* = 8, across 8 RRTT trials. **g** Heatmap analysis of dyadic encounters reveals a predictable social fingerprint pattern of win–loss outcomes in CTR and ELS animals. **h** In both ELS cages and CTR cages, the inter-cage meta-hierarchy assessed in the round-robin (RR) is a strong predictor of intra-cage hierarchy. **i** ELS mice display social subordination in a food competition test when assessing time spent in control of a food pellet after 21 h of food deprivation; CTR *n* = 8, ELS *n* = 8. **j** Total duration for conflict resolution in the tube test was determined as encounter duration in encounters between the three possible dyads: CTR vs CTR (gray), CTR vs ELS (dark red), and ELS vs ELS (red). ELS-ELS dyadic encounters are significantly faster than encounters between ELS–CTR mice or CTR–CTR; CTR *n* = 8 mice, ELS *n* = 8 mice; valid dyadic encounters CTR–CTR *n* = 127, ELS-ELS *n* = 126, CTR–ELS *n* = 506. **k** Encounter duration is independent of the trial outcome in ELS–CTR dyads, as encounter duration is similar when CTR win (CTR_W_–ELS_L_: brown) or lose (CTR_L_–ELS_W_: yellow); CTR *n* = 8 mice, ELS *n* = 8 mice; valid dyadic encounters CTR_W_–ELS_L_ *n* = 392, CTR_L_–ELS_W_ *n* = 113. In a three-chamber test for social behavior, ELS mice display (**l**) normal sociability and (**m**) an increase recognition of social novelty; S_1_—Stranger 1, E—Empty chamber, S_2_—Stranger 2; CTR *n* = 20, ELS *n* = 20. Statistical comparisons were performed using two-way repeated measures ANOVA (for **f**), Spearman correlation (for **h**), unpaired two-tailed *t*-test (for **i**, **l**, **m**), Kruskal–Wallis test with Dunn’s multiple comparisons test (for **j**), Mann–Whitney test (for **k**). Statistical significance was set as *p < 0.05; **p < 0.01; ***p < 0.001. Data are presented as means ± SEM.

We then assessed if maternal separation would impair the ability of animals to form stable social hierarchies (Fig. 2a–c). We performed tube test trials between CTR cage-mates (Fig. 2a, b) or between ELS cage-mates (Fig. 2c) and found that ELS cage-mates still formed linear and transitive hierarchies that remained stable for several days (Fig. 2a–c). Next, we examined whether ELS would affect the dominance rank of animals when these animals were pitted against CTR individuals (Fig. 2d–g). To test this, we designed a round-robin type tournament (Fig. 2d) so that both CTR and ELS mice only encountered unfamiliar CTR and ELS mice from another cage. In this “Round-Robin Tube Test” (RRTT), we observed a striking phenotype of social subordination in ELS mice (two-way ANOVA_CTR_ × _ELS_; *F*_(1,14)_ = 31,31; *p* < 0.0001) (Fig. 2e, f). For each cohort, eight repetitions of a full round-robin tournament were performed to assess the cumulative victories of each animal against every other individual (Fig. 2e). Along these trials, dominance-subordinate relationships with noncage-mate animals did not change over the course of several days (two-way ANOVA_different round-robins_; *F*_(7,98)_ = 0; *p* > 0.9999) (Fig. 2f, g), indicating that a stable meta-hierarchy is achievable even when animals are not housed together. On average, we found that ELS mice lost ~75% of encounters when considering only CTR–ELS encounters (Fig. 2f). At a more granular level, we found that the relationship between CTR and ELS dyads remained surprisingly stable, with 48% of mice remaining undefeated against defined adversaries (Fig. 2g—dark gray and dark red) and only 5% of dyads displaying alternating win–loss encounters (Fig. 2g—white squares). Together, these results strongly suggest ELS produces a stable pattern of social subordination in adult life. Moreover, we repeated this assay and replicated the social subordination phenotype in three other independent cohorts.

Since our animals were naïve to the performance of their cage-mates in the tube test, we also asked if the meta-hierarchy could predict intra-cage social rank. To assess this, we ran cage-mates in tube test encounters, after the completion of the RRTT, and compared their intra-cage hierarchy to the rank predicted from the results of the meta-hierarchy. We found a strong and significant correlation between prediction and experimental data, again suggesting that a meta-hierarchy is a robust procedure to assess social dominance between larger groups of animals (*r*_s_ = 0.8111, *n* = 44, *p* < 0.001) (Fig. 2h).

Finally, to validate the tube test data using a different paradigm, we performed a food competition test between CTR–ELS dyads after mild (21 h) food deprivation. We designed the test so that the animals where fully acclimatized to the test and that a small food pellet could only be held and eaten by one mouse at a time. In this assay, CTR mice spent more time controlling the food pellet when compared with ELS mice (CTR: 60.33 ± 6.067 s, *n* = 14; ELS: 41.78 ± 6.426 s, *n* = 14; *p* = 0.0444) (Fig. 2i), further suggesting submissiveness in social competitions of mice that undergone ELS.

### Faster resolution of social conflicts in animals that experienced ELS

We hypothesized that animals with greater rank difference would resolve the tube test conflict more rapidly [6]. However, while we found that CTR–ELS dyadic encounters did resolve faster than CTR–CTR encounters, our data showed that ELS-ELS encounters displayed the lowest latencies of the three possible encounter types as there was a statistically significant difference between the latencies depending on dyadic combinations (Kruskal–Wallis *H*_(2)_ = 47.54, *p* < 0.0001) with a mean rank of 470 s for CTR × CTR dyads, 382.3 s for CTR × ELS dyads and 280.2 s for ELS × ELS dyads (Fig. 2j). Moreover, we also found that trial duration in CTR–ELS encounters was largely independent of trial outcome (Mann–Whitney *U* = 20380; CTR_wins_ × ELS_loses_: median = 53.44 s, *n* = 393; CTR_loses_ × ELS_wins_: median = 43.9 s, *n* = 113; *p* = 0.1829) (Fig. 2k). We speculated that ELS-ELS encounters resolved fastest due to an enhanced aptitude of these animals to process social cues, which could give them an adaptive advantage in social competitions due to their deficient rearing by allowing a rapid resolution of social conflicts. To test this, we next performed a three-chamber social test for social exploration (Fig. 2l, m). We found no alteration in sociability as measured by the percentage of time CTR and ELS mice spent interacting with a stranger animal *versus* an empty cage (Fig. 2l), since both groups presented equal preference to interact with a stranger animal (Preference for Social_1_, CTR: 59 ± 2.992%, *n* = 20; ELS: 57.72 ± 2.31%; *p* = 0.7367). However, in a second part of the trial, ELS mice spent significantly more time interacting with a novel mouse (Preference for Social_2_, CTR: 54.95 ± 3.787%, *n* = 20; ELS: 67.54 ± 3.413%; *p* = 0.0182) (Fig. 2m). Next, to perform a more challenging task we used a modified version of the three-chamber test that started with two social partners (of the same strain as the test subjects) on each side of the arena (Supplementary Fig. 2A, B). In this test, we observed that CTR mice did not show significant preference for the novel social partner *S*_3_ (CTR: *S*_1–2_ = 43.37 ± 4.782% vs *S*_3_ = 56.63 ± 4.782; *n* = 12; *p* = 0.0625), whereas ELS animals spent significantly more time with the novel partner (ELS: *S*_1–2_ = 40.83 ± 2.917% vs *S*_3_ = 59.17 ± 2.917; *n* = 16; *p* < 0.001) (Supplementary Fig. 2B). These data suggest ELS mice displayed an enhancement in social recognition or the perception of social cues, which may underlie the faster resolution of the tube test conflict.

### Early life adversity induces enhanced inhibitory currents and dendritic atrophy in layer II/III neurons of the mPFC

**Fig. 3:**
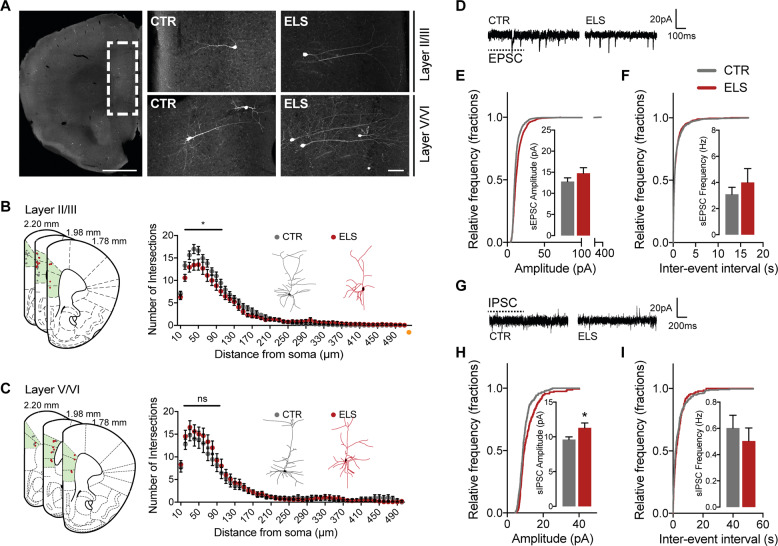
Neuronal hypotrophy and enhanced inhibitory currents in layer II/III pyramidal cells from the mPFC of ELS mice. **a** Peripheral injection of AAV9.hSyn.GFP via the tail vein leads to sparse but strong labeling of neurons in the mouse brain in a Golgi-like pattern. Insets show detail of neurons in layer II/III and V/VI of the mPFC. Scale bar (left) 1 mm, (right) 100 µm. **b** Schematic representation of acquired GFP-labeled mPFC neurons from layer II/III and neuronal complexity of pyramidal neurons in layers II/III in the mPFC is reduced in ELS mice, as assessed by Sholl analysis; CTR *n* = 25/3 neurons/mice, ELS *n* = 17/3 neurons/mice. **c** Schematic representation of acquired GFP-labeled mPFC neurons from layers V/VI; Sholl analysis of pyramidal neurons in this region do not show differences in complexity when comparing neurons from ELS with CTR mice; CTR *n* = 9/3 neurons/mice, ELS *n* = 15/3 neurons/mice. **d** Traces from sEPSC from CTR and ELS adult neurons. Scale bar, 20 pA, 100 ms. Unaltered sEPSC (**e**) amplitude and (**f**) frequency of events in neurons from ELS mice when compared with CTR; cumulative distribution plots for individual excitatory events are plotted and summary data are shown as insets; CTR *n* = 35 cells, ELS *n* = 21 cells. **g** Traces from sIPSC from CTR and ELS adult neurons. Scale bar, 20 pA, 200 ms. Pyramidal neurons in the mPFC display (**h**) an enhancement in inhibitory currents, but (**i**) no change in the frequency of inhibitory events; cumulative distribution plots for individual inhibitory events are plotted and summary data are shown as insets; CTR *n* = 36 cells, ELS *n* = 20 cells. Statistical comparisons were performed using two-way repeated measures ANOVA (for **b** and **c**) or two-tailed unpaired *t*-test. Significance was set at, *p < 0.05. Data in bar graphs are presented as means ± SEM.

To investigate if cellular alterations occur upon perturbed rearing conditions in the early postnatal period, we collected information on morphological and electrophysiological parameters from pyramidal neurons in the mPFC of adult mice. We chose to investigate the mPFC since this region is: (1) strongly targeted by the glucocorticoid system [8], (2) responsible for top-down control of other brain systems according to the animals’ internal state and intention [22], and (3) has been strongly implicated in the regulation of social hierarchy behaviors [6]. To investigate neuronal morphology changes in ELS mice, we performed peripheral injection of adeno-associated viral particles containing a GFP construct (AAV9.hSyn.GFP) via the tail vein to achieve a Golgi-like labeling of neurons in the whole mouse brain (see “Materials and methods” section and [17]). We then performed a reconstruction of neurons using confocal microscopy and assessed neuronal arborization and complexity via Sholl analysis. Interestingly, we found a specific defect in the arborization of layer II/III pyramidal neurons of ELS mice (two-way RM ANOVA_10–100µm: CTR×ELS_ *F*_(1,40)_ = 4.552; *p* = 0.0391) (Fig. 3a–c). However, no significant changes in spine density were present in these neurons (Supplementary Fig. 3). Moreover, we found no significant alterations in the dendritic arbors of neurons from layer V/VI (Fig. 3c), nor in the dendritic arborization of layer II/III neurons in frontal motor and somatosensorial cortex (Supplementary Fig. 4). These results suggest there is both a region- and layer-specific alteration, which is in-line with that observed following chronic stress, which results in localized dendric remodeling [10, 23]. Moreover, this demonstrates that early life rearing conditions strongly influence cortical network organization, specifically affecting the morphology of pyramidal neurons in layers II/III of the mPFC.

We became interested in understanding if there were functional alterations in synaptic activity in the mPFC region. To this end, we performed whole-cell patch clamp in acute slices from adult (4-month old) CTR and ELS mice, using a modified slice recovery method [18]. We measured spontaneous network activity of both excitatory and inhibitory postsynaptic currents (sEPSCs and sIPSCs) in layer II/III pyramidal neurons (Fig. 3d–i). While we found no significant differences in sEPSC amplitude or frequency in adult animals (Fig. 3d–f), there was a significant increase in sIPSC amplitude in neurons from adult ELS mice (CTR: 9.607 ± 0.3894 pA, *n* = 36; ELS: 11.30 ± 0.6958, *n* = 20; *p* = 0.0253) (Fig. 3g–i). These results are in line with recent findings suggesting alterations in inhibitory neuron networks that occur as a consequence of ELS [24, 25].

In order to assess if these morphological and functional differences stemmed from persistent increases in corticosterone levels or if ELS mice were hyperreactive to stressful insults, we determined the concentration of corticosterone under basal conditions (between days of experimentation) and immediately following tube test encounters (Supplementary Fig. 5). While the tube test did significantly increase circulating corticosterone levels, there were no changes between CTR and ELS male mice before or immediately after running a tube test trial (Supplementary Fig. 5). This suggests that physiological and behavioral alterations are not necessarily propagated via a dysfunctional increased release of corticosterone in adult ELS mice. In addition, we found no evidence for significant alterations in the expression levels of corticosteroid receptors (see RNAseq data below and Supplementary material).

### Global gene expression and functional analysis reveal *Npy1r* is overexpressed in the prefrontal cortex of ELS mice

**Fig. 4:**
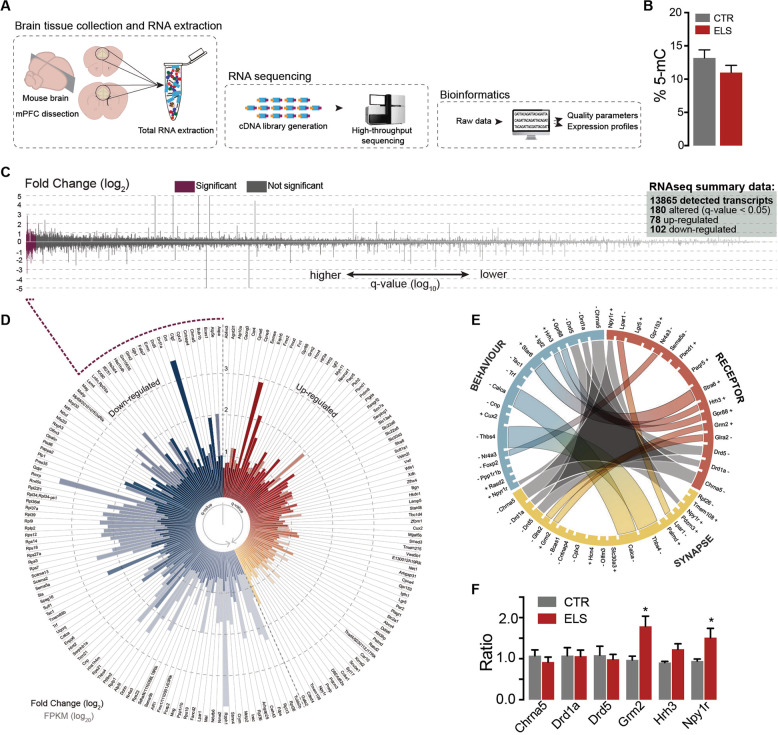
Global alterations in gene expression in the mPFC of ELS mice. **a** Schematic representation of the experimental procedure of mPFC RNAseq data collection. **b** Global DNA methylation levels are not significantly altered in the mPFC of adult male mice; CTR *n* = 7, ELS *n* = 9. **c** Normalized trace representation of fold change (log_2_ on *y* axis) and *q* value (log_10_ on *x* axis) for all transcripts detected in the RNAseq in CTR and ELS animals (total of 13,865 transcripts); significantly altered transcripts are represented in purple as *q* < 0.05 in FDR adjusted *p* values; CTR *n* = 3, ELS *n* = 3. **d** Circos plot display of upregulated transcripts in red (total of 78) and downregulated transcripts in blue (total of 102) in ELS mice compared with CTR; solid bars represent fold change (log_2_) and transparent portion of each bar represents transcript abundance in Fragments Per Kilobase Million (FPKM in log_10_). **e** Chord diagram highlighting genes belonging to the Gene Ontology categories, “Biological Process—Behavior,” “Cellular component—Synapse,” and “Molecular Function—Receptor Activity”; genes belonging to all three categories are connected by gray cords. **f** Validation by qRT-PCR of receptor channels involved in behavioral regulation; CTR *n* = 5–13, ELS *n* = 6–18. Statistical significance was set at, *p < 0.05 with FDR correction. Data in (**b**) and (**f**) are presented as means ± SEM.

Next, we aimed to assess global gene expression changes in the mPFC of ELS mice (Fig. 4a). We tested for overall DNA methylation changes, but found no significant alteration between groups (Fig. 4b). Using RNAseq (Fig. 4a and Supplementary Fig. 6), we detected a total of 13,865 transcripts, from which 180 displayed altered expression levels in the mPFC of ELS mice (Fig. 4c). From these, 78 transcripts were upregulated and 102 downregulated as illustrated in Fig. 4d and Supplementary Table 1. Consistently to what has been previously found for primate and rodent models of neonatal stress, we observed alterations in genes related to myelination [26, 27], such as *Mag*, *Mog*, *Mal*, *Opalin*, and *Plp1*, as well as alterations in targets proposed to be involved in chronic stress, such as *Grm2* [28, 29]. Due to our behavioral and electrophysiological findings, we next decided to focus on the intersection between three gene ontology groups, “Biological Process—*Behavior*,” “Cellular component—*Synapse*,” and “Molecular Function—*Receptor Activity*” (Fig. 4e and Supplementary Tables 2–7), selecting for this analysis the following receptors for downstream validation: *Chrna5*, *Drd1a, Drd5, Grm2, Hrh3*, and *Npy1r* (Fig. 4e). Next, using a larger number of animals produced from different ELS cohorts, we validated which of these alterations were more saliently present in the stress model. From our target genes we found consistent increases in the expression of *Grm2* and *Npy1r* in the mPFC of ELS mice (one-way ANOVA *F*_(11,129)_ = 2.348; Post-hoc multiple comparisons FDR < 0.05) (Fig. 4f).

Interestingly, NPY1R is heavily expressed in the frontal cortex and NPY is co-released with GABA by a subgroup of cortical interneurons [30]. While the physiological influence of NPY in cortical excitability has been complex to dissect, recent evidences strongly point to a role of this receptor in amplifying inhibitory inputs onto pyramidal neurons [31, 32]. Moreover, NPY1R is known to play a role in biological functions related to anxiety and feeding [33]. Interestingly, even in lower organisms, the *Npy1r*-orthologue can impact both social and foraging behaviors, with a naturally occurring point-mutation in this gene defining if a specific strain exhibits solitary or social feeding behavior [34].

**Fig. 5:**
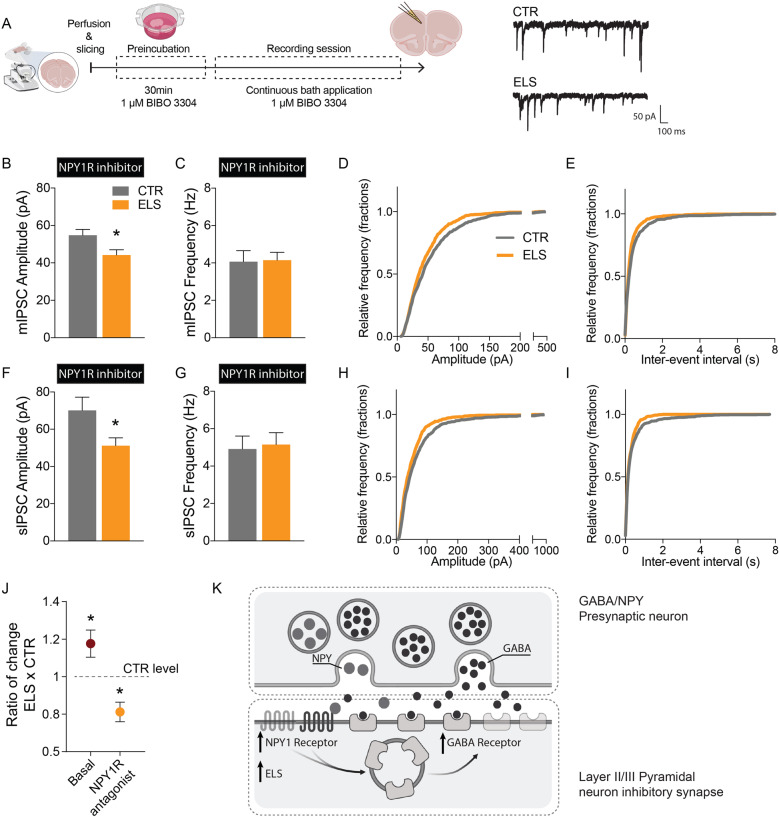
Inhibiting NPY1R leads to a marked decrease in inhibitory currents in pyramidal neurons from adult ELS mice. **a** Experimental setup (left) and representative traces (right) from inhibitory postsynaptic currents (mIPSC) from CTR and ELS neurons. To record chloride currents as salient inward currents, in this experiment the composition of the intracellular solution was altered to include high intracellular Cl^−^ concentration, while AMPA and NMDA receptors were blocked (see “Materials and methods” section). Scale bar, 50 pA, 100 ms. Reduced amplitude of mIPSC (**b**), but no changes to the frequency of events (**c**). Cumulative distribution plots for individual inhibitory events plotted as amplitude (**d**) and inter-event interval (**e**); CTR *n* = 27/5 cells/mice, ELS *n* = 19/4 cells/mice. Reduced amplitude of sIPSC (**f**), but no changes to the frequency of events (**g**). Cumulative distribution plots for individual inhibitory events plotted as amplitude (**h**) and inter-event interval (**i**); CTR *n* = 28/5 cells, ELS *n* = 18/4 cells. **j** Summary data from spontaneous inhibitory currents in ELS mice normalized to CTR, under basal conditions (see Fig. 4) and in the presence of the NPY1R antagonist. **k** Schematic representation of a potential mechanism where postsynaptic NPY1R leads to increased GABA_A_ receptor membrane insertion. Statistical comparisons were performed using two-tailed unpaired *t*-test. Significance was set at, *p < 0.05. Data in bar graphs are presented as means ± SEM.

Taking the above into consideration, we probed the relative influence of NPYergic tone on overall inhibition in pyramidal neurons in the mPFC of CTR and ELS mice. To assess this, we preincubated acute slices from 4-month-old animals with 1 µM BIBO 3304 trifluoroacetate (a selective NPY1 receptor antagonist) to discount the influence of NPY1R contribution toward inhibitory currents and recorded mIPSCs and sIPSCs. In this paradigm we recorded the GABAergic component as inward currents (see “Materials and methods” section) and observed a striking reduction in the amplitude of GABA-mediated currents in neurons from ELS mice both in spontaneous (CTR: 70.16 ± 7.097, *n* = 27; ELS: 51.2 ± 4.166, *n* = 19; *p* < 0.05) and miniature events (CTR: 54.74 ± 3.144, *n* = 27; ELS: 44.22 ± 2.847, *n* = 18; *p* < 0.05) when compared with CTR mice (Fig. 5). These data strongly suggest that not only is the inhibitory component increase in ELS mice being mediated by an upregulation in NPY1R, it suggests that this form of stress skewed mPFC micro-circuitry toward a greater NPYergic activation, since blocking NPY1 receptors reduced inhibitory currents significantly below control levels (Fig. 5j, k).

## Discussion

Several studies have demonstrated that during early childhood, brain regions such as the hippocampus, the amygdala, and the mPFC are particularly sensitive to the effects of chronic stress [5, 35]. In fact, maternal separation during this period has a remarkable influence in adult life [21, 36]. In this work we probed the molecular, cellular, and circuit alterations occurring in the mPFC of ELS animals and determined that traumatic maternal separation alters adult behavior and promotes the modulation of social strategies in male mice. We found no alterations in serum levels of corticosterone between control and ELS adult mice, before or after social hierarchy tests, suggesting that our ELS does not necessarily produce a maladaptive stress response or hyperreactivity in the HPA axis in adulthood.

We found a mild change in depressive-like behavior and observed a reduction in latency for the animals to become immobile in the forced swimming test, but no overall changes in total time spent in immobility. Since our results are sufficiently powered, our data suggest that the depressive-like behavior in male rodents may not be a principal consequence of maternal deprivation. This would be in line with a phenotypical manifestation of depression that is incompletely penetrant, prone to surface [21, 37] or not [38], depending on experimental variables or additional triggers [39].

From an ethological perspective, male mice experiencing hunger and improper rearing conditions during early life periods may be handicapped in future physical disputes over resources within the group setting. Thus, our findings of enhanced discrimination of social targets, social subordinance, and a faster resolution of social conflicts presented by ELS mice, in addition to their increase in foraging-like behaviors, point toward behavioral adaptations that could promote disengagement from competitions with higher ranked males. Therefore, we propose that ELS promotes an adaptation that induces foraging and reduced competition within the social group. As summarized by Wilson from the works of Christian [40] and Calhoun [41], “emigration and egress are tools of juveniles and subordinates” [42]. Furthermore, and as an extrapolation of our hypothesis, if male and female rodents provide nonequivalent contribution to overall group fitness under conditions of scarcity and stress, it would follow that behavioral adaptations also differ. Indeed, this is in line with several reports showing sexual dimorphic response as a consequence of early postnatal stress, whereby females show greater resilience to ELS [43, 44]. Future work should aim at understanding the sex-specific circuit level changes that promote different strategies in animal behavior.

At the genetic level, selective breeding programs have demonstrated that traits regulating social dominance may be rapidly enhanced in the span of only a few generations [45, 46], and genetic association studies have also suggested that traits influencing social dominance may be inherited [47]. However, the identification of individual genes and direct assessment of their impact on the cellular and synaptic network activity responsible for social dominance has not been widely scrutinized [47, 48]. Some examples include the serotonin transporter *Slc6a4* knockout mice [49] and the dopamine transporter knockouts [50]. In addition, there are dysfunctional dominance behaviors linked to synaptic proteins such as Shank and Synapsin [51–53].

At the same time, there are also evidences that implicate environmental conditions, such as stress and “rank inheritance” leading to epigenetic alterations that affect dominance [54, 55]. In addition, the prior history of each animal and his winning/losing experience also play a role in future encounters [56, 57]. With this in mind, we designed our RRTT trials to ensure there are CTR *versus* CTR trials, to mitigate the possibility of the “winner effect” [57], and guarantee that some CTR mice also lose before first encountering ELS mice. Importantly, our data show that there is a stable dominance gradient persisting for several weeks, even when mice are not co-habiting the same cage. This “meta-hierarchy” allowed us to robustly predict the intra-cage rank of male mice before they competed against cage-mates. This implies that dominance is not merely imparted via physical contests in the home cage, and that the position in a meta-hierarchy is transitive to the broad group of animals. However, the question of how mice transmit, recognize, and calculate social dominance cues in a tube test is not well understood, and may potentially arise from a combination of procedural behavioral interactions (e.g. push, resist, retreat) [14], odors [58], or other sensorial communications that may signal rank (e.g. whisker position) [59].

After a broad analysis of altered transcripts in the mPFC of ELS mice we focused our attention on *Npy1r*, a G-protein coupled receptor that has been linked to maternal care and anxiety [60], foraging behaviors [61], and regulation of social behaviors [34]. From our findings via RNAseq analysis of the mPFC, we postulated that *Npy1r* could mediate behavioral adaptations linked to subordinance and increased risk-taking behavior.

At the synaptic level, our observation of increased amplitude of inhibitory postsynaptic currents is in line with evidences linking NPY1R as a potentiator of GABA-mediated currents [30, 62]. We also speculate that the most parsimonious explanation for the results in increased inhibitory tone are due to enhanced NPY1R activity, as these data are supported by RNAseq, qRT-PCR, and pharmacological data. However, another contributing factor could be linked to the location of NPYergic activity, since in the cortex, NPY neurons have been proposed to sit close to cortical layer I [63]. Therefore, the observation of neuronal hypotrophy in layer II/III pyramidal neurons, could contribute electrotonically to an enhancement of NPYergic activity if these synapses are now occurring closer to the soma. Also, our recordings from adult mice, provides a view on a mature circuitry, which complements evidence that normally focus on animals of younger ages. This is important to consider, since prenatal insults have been shown to lead to age–dependent, biphasic changes in the inhibitory system [64].

Anxiety levels, in both mice and humans, have also been linked to social dominance [65–67]. These observations directly intersect with recognized anxiolytic and antistress modulatory actions of NPY [68, 69]. Therefore, future work should aim at dissecting if NPYergic signaling in the mPFC is necessary and sufficient to modulate social dominant behaviors, and explore the role of this neuropeptide in frontal cortico-striatal circuitry, a key pathway in social dominance [65, 67]. Another important aspect to also consider is if the changes in NPYergic activity are a compensatory mechanism, and how specifically bolstering/inhibiting this receptor in the frontal cortex modulates behavior in the context of ELS.

Altogether, this work identifies an adaptive behavioral strategy in animals that are subjected to ELS and implicates NPY1R as a regulator of critical changes in mPFC circuitry. This receptor and the control of inhibitory synaptic activity are potential targets to better understand the effects of chronic stress and of persistent social subordination in adult life.

## Funding and disclosure

This research was supported by the Portuguese Foundation for Science and Technology (FCT) Investigator Program IF/00812/2012, FCT grant POCI-01-0145-FEDER-016682, Marie Curie Career Integration Grant (#618525 to JP) and Marie Curie Individual Fellowhip (#799164 to CMS), NARSAD Young Investigator Grant from the Brain & Behavior Research Foundation (#20733), and Bial Foundation Grant 266/2016 (to JP) and 264/2016 (to ALC). We thank the support from FEDER/COMPETE institutional funds POCI-01-0145-FEDER-007440, BrainHealth 2020 CENTRO-01-0145-FEDER-000008; fellowships SFRH/BD/51961/2012 (to LOF), SFRH/BD/105878/2014 (MIT-Portugal to MJC), SFRH/BPD/120611/2016 (to JRG), SFRH/BD/144224/2019 (to PAF),  SFRH/BD/144875/2019 (to JC) and SFRH/BD/51958/2012 (to ME). The authors declare no conflict of interest.

## Supplementary information

Supplementary Material

Supplementary Figure 1

Supplementary Figure 2

Supplementary Figure 3

Supplementary Figure 4

Supplementary Figure 5

Supplementary Figure 6
