# Supplementary material for: Social subordination induced by early life adversity rewires inhibitory control of the prefrontal cortex via enhanced Npy1r signaling
Source: Neuropsychopharmacology. 2020 Jun 3;45(9):1438–47. doi: 10.1038/s41386-020-0727-7 (PMC7360628; doi:10.1038/s41386-020-0727-7)
Supplement: Supplementary file 1 — Supplementary Material [file 41386_2020_727_MOESM1_ESM.pdf]

Supplemental Figures and Tables:

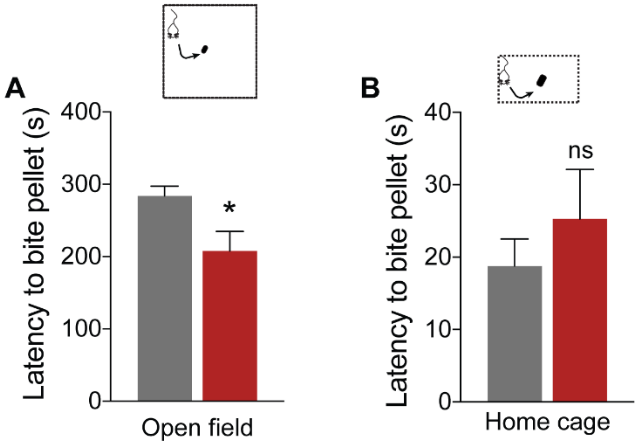

Supplementary Figure 1 | ELS reduces latency to bite food pellet in an open field. (**A**) In an open field test ELS mice display lower latency to bite a food pellet when compare to CTR, while in (**B**) the home cage this difference is no longer observed.; CTR n=7, ELS n=7. Statistical comparisons were performed using two-tailed Mann-Whitney test. Statistical significance was set as *p<0.05. Data are presented as means ± SEM.

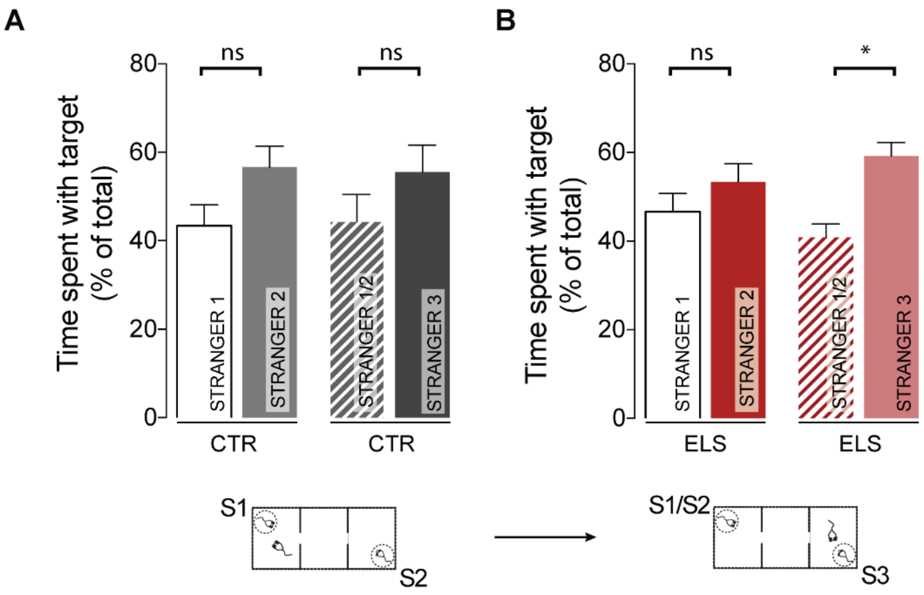

Supplementary Figure 2 | ELS induces enhanced recognition of social partners in a modified 3-chamber test. A-B, An assay using a modified three-chamber test for social recognition starts with two social partners and, in a second part, one of the animals is substituted by a novel stranger mouse (S3 – Stranger mice). In this task CTR mice (**A**) do not show a preference between Stranger 1 and Stranger 2, or between Stranger 1/2 and Stranger 3; ELS mice (**B**) show an increase in the percentage of time in interaction with the novel partner; CTR n=12, ELS n=16. Statistical comparisons were performed using two-tailed t-tests. Statistical significance was set as *p<0.05. Data are presented as means ± SEM.

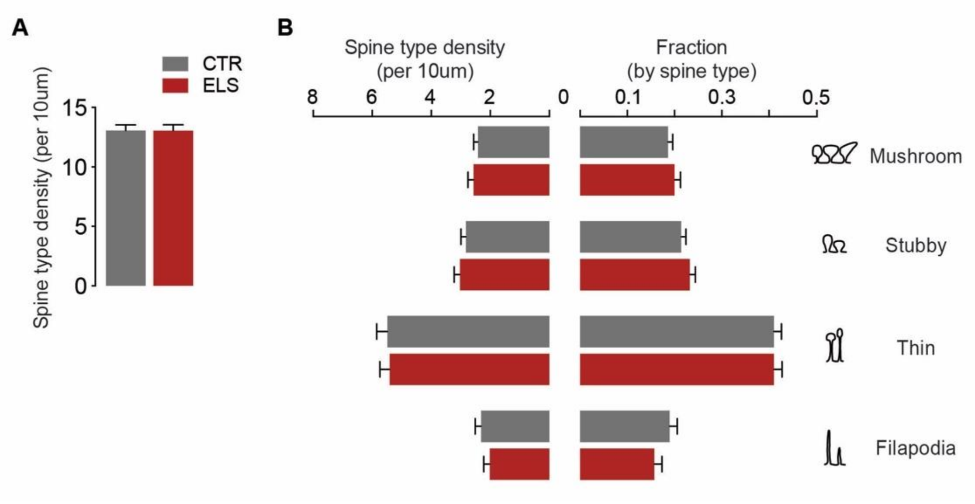

Supplementary Figure 3 | Spine density is not significantly altered in layer II/III pyramidal cells from the mPFC of adult ELS mice. A, No changes were observed in spine density when comparing CTR and ELS secondary dendrites of layer II/III neurons in the mPFC. **B**, When analyzing spine types by density (left) and fraction (right), no significant changes were observed; CTR n=59, ELS n=47 dendrites. Data is presented as means ± SEM.

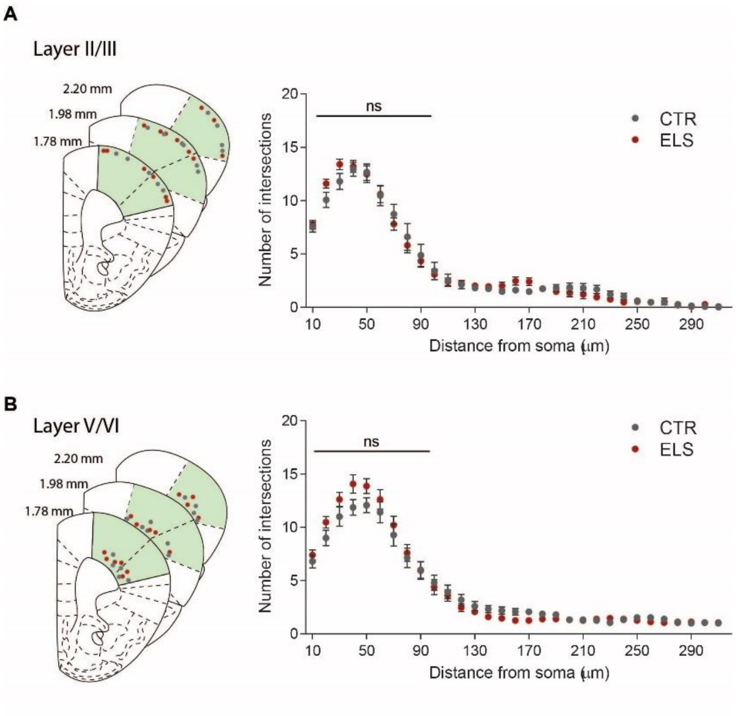

Supplementary Figure 4 | Neuronal morphology of layer II/III and V/VI pyramidal cells from the somatosensorial and motor cortex are not perturbed by ELS mice. A, Schematic representation of acquired GFP-labeled neurons from layer II/III and Sholl analysis of neuronal complexity; CTR n=15, ELS n=15. **B**, Schematic representation of acquired GFP-labeled mPFC neurons from layers V/VI and Sholl analysis of neuronal complexity; CTR n=15, ELS n=15. Statistical comparisons were performed using two-way repeated measures ANOVA and significance was set at, * $p<0.05$. Data are presented as means ± SEM.

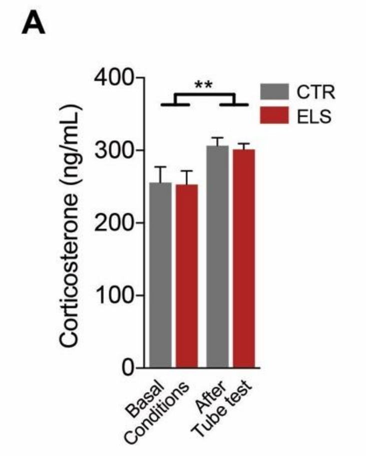

Supplementary Figure 5 | ELS does not alter serum corticosterone levels. A, Serum corticosterone levels in ELS and control adult male mice assessed under basal conditions and after a round-robin tube test trial, CTR n=7, ELS n=8. Statistical comparisons were performed using two-way ANOVA. Statistical significance was set as **p<0.01. Data are presented as means ± SEM.

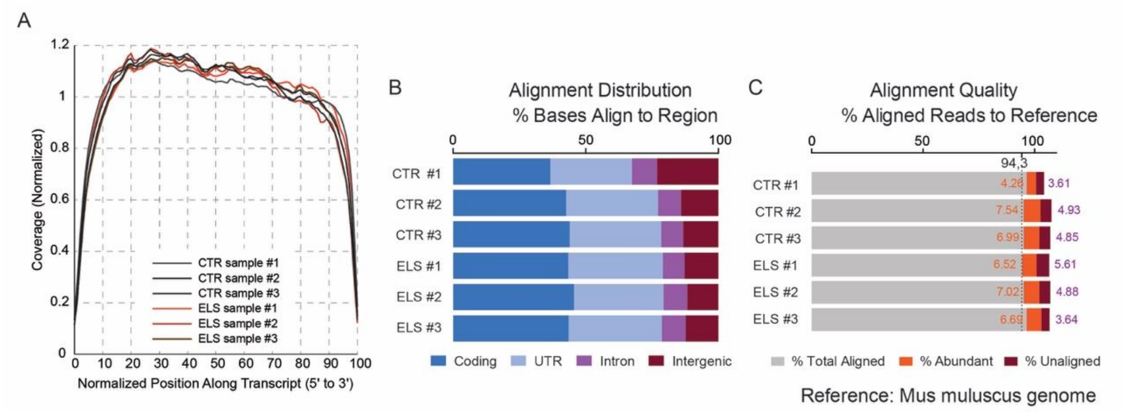

Supplementary Figure 6 | Quality control of RNAseq and transcript alignment to the reference genome. A, Transcript coverage normalized by position along the transcript (percentile). **B**, RNAseq alignment distribution as histogram graphs of the number of bases by genomic region (coding, UTR, intron or intragenic). **C**, Alignment quality as percentage of reads passing quality filter that aligned (% Total Aligned, grey); as percentage of reads that align to abundant transcripts, such as mitochondrial and ribosomal sequences (% Abundant; orange); and as percentage of reads that do not align to the reference genome (% Unaligned, purple). UTR; Untranslated region.

Supplementary Materials and Methods

Preparation of brain slices for electrophysiology. Acute prefrontal cortex slices were prepared from CTR and ELS mice (4-5-month-old mice) by an experimentalist blinded to the phenotype of the animals. Before brain dissection, mice were deeply anesthetized with isoflurane and perfused with oxygenated (95%:5% O_2:CO_2 mix) N-Methyl-D-Glucamine-enriched artificial cerebrospinal fluid (NMDG-aCSF) containing (in mM): NMDG 198.86, KCl 2.5, $NaHCO_3$ 26, $NaH_2PO_4.2H_2O$ 1.25, Glucose 12.5, Thiourea 2, L-ascorbic acid 1, Na-pyruvate 3, $MgSO_4$ 10 and $CaCl_2$ 0.5. The brain was quickly removed and glued to a vibratome support filled with ice-cold, oxygenated aCSF. Coronal prefrontal cortex slices of 300 µm were obtained using a vibratome (Leica VT1200s, Leica Microsystems, USA) and immediately recovered at 32 °C for 8 min in NMDG-aCSF. Slices were then moved to a holding chamber that contained oxygenated artificial cerebrospinal fluid (aCSF) with the composition (in mM): NaCl 120, KCl 2.5, $NaHCO_3$ 24, $NaH_2PO_4.2H_2O$ 1.25, Glucose 12.5, $MgSO_4$ 2 and $CaCl_2$ 2. Before recording, the slices were placed for at least 1 h at room temperature, in oxygenated aCSF. Slices where finally moved to the recording chamber and perfused with oxygenated aCSF (2 to 3 mL/min) at 25°C.

The osmolarity of all solutions was adjusted to 300-310 mOsm except for the potassium-gluconate based internal solution (K-int, see below) which was adjusted to 295–298 mOsm. The pH of all solutions was adjusted to 7.4 with HCl, except for the K-internal (adjusted with KOH).

Whole-cell patch clamp. Prefrontal cortex pyramidal neurons were identified under infrared-differential interference contrast (IR-DIC) visualization. Cells were patched with borosilicate glass recording electrodes (3-5 MΩ; Science Products) filed with an internal solution. To record spontaneous excitatory and inhibitory postsynaptic currents (sEPSCs and sIPSCs) K-int internal solution was used, containing (in mM): K-gluconate 145, HEPES 10, EGTA 1, Na-phosphocreatine 10, ATP magnesium salt 2, GTP sodium salt 0.3 and $MgCl_2$. The recorded neurons were voltage-clamped at -55mV, to simultaneously record sEPSC and sIPSC.

In the experiments used to record only inhibitory postsynaptic currents (sIPSCs and mIPSCs) CsCl internal solution was used, containing (in mM): CsCl 120, HEPES 2, Lidocaine N-ethyl chloride 5, ATP sodium salt 2, GTP sodium salt 0.4 and $MgCl_2$ 2. The recorded neurons were voltage-clamped at -70mV. In order to isolate sIPSCs the following drugs were added to the recording aCSF (in µM): AP-5 25, CNQX 10. To isolate mIPSCs, the same drugs were used, with the addition 0.5 µM of tetrodotoxin. For mIPSC and sIPSC recordings under antagonism of NPY1R receptors, slices were kept at least 30 min in oxygenated aCSF containing 1 µM BIBO 3304 trifluoroacetate, a highly selective NPY1R receptor antagonist. The recordings were performed in aCSF containing the same concentration of the drug. The first 30 events of each recording were chosen for analysis. Criteria for acceptance of cells was determined as a stable Ra under 25 MΩ. Recordings were filtered at 2 kHz and digitized at 20 kHz.

Data was acquired with a Multiclamp 700B amplifier and Digidata 1550A (Molecular Devices Corporation) and analysed using Clampfit 10.7 software (Axon Instruments).

Forced swimming test. The forced swimming test mice was performed in a 2 L glass beaker (19 cm high with a diameter of 13 cm) filled with water (at 18 ± 1 °C) up to 1,5 L. Testing started by introducing subjects in a beaker and behaviors were recorded for 6 min. Indirect and homogeneous illumination of the room was provided by white LED lamps at 20 lx. Latency to stop swimming and total time spent immobile were evaluated. Immobility was defined as the lack of motion of the entire body, except small movements to keep mouse head above water.

Elevated plus maze. The elevated plus maze consisted of a plus-shape apparatus with two open arms (without walls) and two closed arms (with walls), each one with 30 x 5 cm. Mice were placed at the center of the apparatus, facing one of the closed arms, and behavior was video-tracked automatically using Ethovision XT (Noldus, Netherlands) for 10 min. Direct illumination of the maze was provided by white LED lamps with open arms illuminated at approximately 300 lx. Time spent in the open arms and latency to enter an open arm were quantified automatically.

Open field. The open field consisted of an opaque arena (40 x 40 x 30 cm). Mice were placed at one corner of the apparatus and locomotor behavior was recorded for 1 h using Ethovision XT (Noldus, Netherlands). Indirect and homogeneous illumination of the room was provided by white LED lamps set at 100 lx. Time spent in the center zone (15 x15 cm) and total distance travelled were evaluated automatically.

Three-chamber social interaction test. The three-chamber arena was from Stoelting (Stoelting, Ireland). Animals were tested for voluntary social interaction as previously described [1]. The assay consisted of three sessions: the first session began with a 20-min habituation period during which the subject mouse freely explored all three chambers; next, the mouse was confined to the centre chamber and an empty wire cage (Empty - 'E') and a cage with an unfamiliar mouse (Stranger 1 - 'S1') were introduced to the side-chambers; in the second session, the subject mouse was then allowed to freely explore all three chambers for 10 min. Following the 10-min session, the animal remained in the chamber for an extra 10 min (post-test) to better acquire the identification cues from the 'S1' animal. Before the third and last session, the subject mouse was gently guided to the centre chamber while the empty wire cage was replaced with a caged stimulus mouse (Stranger 2 - 'S2'). In the last session, the subject mouse was then left explored all three chambers for 10 min. Stimulus mice were males of the same age and previously habituated to the wire cages. The positions of the empty cage and 'S1' were alternated between tests. Time spent in close proximity, distance travelled, and heat maps were calculated using the automated software Ethovison XT (Noldus, Netherland).

The modified 3-partner social test was performed as above but started with 2 social partners. Afterwards, one partner was randomly replaced by a novel stranger animal (Stranger 3) in the second part of the trial. Indirect and homogeneous illumination of the room was provided by white LED lamps at 50 lx.

Tube test. The tube test was performed in a transparent plexiglass tube, 33 cm long with an inner diameter of 3 cm. Acrylic ramps were placed to allow the animals easy access and retreat back from the tube. Testing started by introducing two different age-matched subjects to the edges of the tube. Testing ended as soon as one of the subjects had all paws outside of the tube for at least 4-seconds. All animals were weighed before each round. Indirect and homogeneous illumination of the room was provided by white LED lamps at 20 lx. For round-robin tournaments a pseudo-random encounter table was designed to fulfill the following criteria: a) encounters were performed only between unknown and non-cage-mate individuals; b) two cages of each group (total of four cages) were always included to insure that most animals from both groups experienced both victory and defeat (i.e. the CTR-CTR trials provided CTR winners and CTR losers and similar for ELS animals); c) each individual was tested against all individuals; d) the number of encounters between tested individuals was maintained as close as possible at each dyadic encounter to ensure individuals that met inside of the tube had similar experience and e) entrances from each end of the tube were counterbalanced.

Food competition test. The food competition test was performed in clean cages. Animals were food deprived for 21 h. On the testing day, dyads of mice were allowed to compete for a food pellet for 5 min. Indirect and homogeneous illumination of the room was provided by white LED lamps at 20 lx. The time spent grabbing the food pellet was scored manually by an experimentalist blind to the animals' treatment using the Observer XT 12.0 (Noldus Information Technology).

***In vivo* morphology analysis of neurons and spines in the mPFC.** To achieve sparse, Golgi-like labelling of neurons in the CNS, we performed injections in the tail vein of 4-month-old animals with 5 µL of AAV9.Syn.eGFP.WPRE.bGH (titter of 8.88×10^{12}, from Penn Vector Core, University of Pennsylvania, PA) diluted in sterile PBS to a final volume of 100 µL. Six weeks post-injection, animals were sacrificed, the brain collected and processed for neuronal imaging. Briefly, mice were anesthetized with isoflurane and perfused transcardially with PBS followed by 4% PFA in PBS, pH 7.4. Whole brain was dissected and post-fixed in 4% PFA for 24 h, followed by transfer to a 30% sucrose solution in PBS. Serial coronal sections of 100 µm were collected using a vibratome (Leica VT1200s, Leica Microsystems, USA) and mounted in gelatinized slides using Vectashield with DAPI (Vector Laboratories) as mounting medium. Slides were stored at 4ºC and protected from light until further analysis. Images of pyramidal neurons from the mPFC were acquired in an LSM 710 Confocal microscope (Zeiss, Germany) with a Plan Apochromat 20x/0.8 DICII lens. Each image consisted of a stack of images taken through the z-plane of the section. Confocal microscope settings were kept the same for all scans in each experiment. Neurons expressing GFP were chosen from layer II/III and V/VI in the mPFC. Neuronal tracing reconstruction was performed using

Neurolucida (MBF Bioscience). Spines on secondary dendrites of mPFC neurons were acquired using a LSM 710 Confocal microscope (Zeiss, Germany) with a Plan Apochromat 63x/1.4 NA oil objective. Spine density and size analysis was performed using Neurolucida (MBF Bioscience). Each spine was included in one of four categories: filopodia (spines without a defined head), stubby (spines without a defined neck), thin (with a neck and head diameter smaller than double the width of the neck) and mushroom (with a neck and head diameter larger than double the width of the neck). Sholl analysis was performed using NeuroExplorer software (MBF Bioscience). Experiments were performed blind to animals´ treatment during both image acquisition and image analysis.

Preparation of DNA and RNA samples. Before brain dissection, 4-5-month-old mice were deeply anesthetized with isoflurane and rapidly sacrificed by decapitation. The brain was sliced using a cooled metal matrix (BrainTree) and the slice containg the mPFC was collected using tissue punches (<10 mg of tissue per animal), the tissue was then flash frozen in liquid nitrogen and stored at –80°C. Genomic DNA and total RNA were simultaneously purified using the AllPrep DNA/RNA/miRNA Universal Kit (Qiagen), and according to the manufacturer's protocol. Final concentrations were measured with a Nanodrop 2000 spectrometer (Thermo Scientific) and samples stored at –20°C (DNA) or –80°C (RNA).

DNA methylation assay. The total levels of 5-methylcytosine (5-mC) were measured using the 5-mC DNA ELISA kit (Zymo Research) according to the manufacturer's instructions. A total of 100 ng of genomic DNA were added to the coating buffer, to a final volume of 100 µL, and then denatured at 98°C for 5 min. Seven standard samples with different percentages of 5-mC DNA (from 0% to 100%) were prepared and assayed in parallel with the tested samples. Standards and samples were measured in duplicate at 405 nm, using a SpectraMax Gemini EM fluorimeter (Molecular Devices). A standard curve was obtained by a second-order logarithmic regression and used to determine the percentage of 5-mC for tested DNA samples.

RNA sequencing and transcriptomic analysis. Before RNA sequencing, the concentration and quality of total RNA samples were determined using the Experion RNA StdSens Analysis Kit (BioRad). Ribozero RNA libraries were prepared from RNA samples and used for RNA sequencing (100 bp Single Read Sequencing, ~20 million reads/sample) with Illumina 2500 HiSeq platform. RNA libraries and sequencing were performed by the Genomics Core Facility from Icahn School of Medicine at Mount Sinai (NY, US).

RNA sequencing data analysis was performed on the Illumina BaseSpace platform (Illumina), and based on the protocol established by Trapnell et al.[2]. Reads for each treatment group were aligned to the *Mus musculus* genome (genome reference: Mus musculus UCSC mm10), and the resulting alignment files were used to create a transcriptome assembly for each experimental condition. The two resulting assemblies were merged, allowing a uniform determination of the transcript expression levels for both

groups. Based on the reads and the merged assembly, expression levels changes, provided as the fold change (in log_2), and statistical significance were calculated. Other analysis, such as false discovery rate (FDR), correct q-values, and assembly of transcript attributes, including common names and locations in the genome, were also performed using BaseSpace platform.

qRT-PCR. RNA samples (250 ng) were converted to complementary DNA using the NZY First-Strand cDNA Synthesis Kit, and following the manufacturers protocol (NZYTech). Contamination with genomic DNA was avoided with (1) on-column DNase I treatment during RNA extraction and (2) qRT-qPCR primers complementary to cross adjacent exons. The synthesized complementary DNA was stored at –20 °C.

The qRT-PCR was performed in an iQ5 thermocycler (BioRad) and primers were designed with Beacon Designer 7 software. Chrna5 FW 5'-CAGTTAGTGGACGTGGATGA-3', Chrna5RV 5'-CCACCATAGTCATCAGGATTCC-3', Drd1a FW 5'-TCTCAGTCACTTTTCGGGGA-3', Drd1a RV 5'-CAGATCGGGCATTTGGAGAG-3', Drd5 FW 5'-CTATTTCCAGACCCTTCCGC-3', Drd5 RV 5'-AGTTGGACCGGGATAAAGGA-3', Grm2 FW 5'-CTCCAGTGATTATCGGGTGC-3', Grm2 RV 5'-AGGATAATGTGCAGCTTGGG-3', Hprt FW 5'-CCCTGGTTAAGCAGTACAGC-3', Hprt RV 5'-ATCCAACAAAGTCTGGCCTG-3', Hrh3 FW 5'-GTCAGTCACTCGAGCTGTCT-3', Hrh3 RV 5'-AGGCCCATACAGCAGGAAG-3', Npy1r FW TGCTACTTCAAGATATACATTCGC-3', Npy1r RV 5'-GGAGAGCAGCATGATGTTGA-3'. Primer specificity was verified by the melting curve shape, peak temperature and the amplification of a single product with the expected size.

A master mix was prepared with (1) iQ SYBR® Green Supermix (BioRad) (5 µL per well), (2) both primers (0.5 µL per well), and (3) complementary DNA (4 µL, diluted 1:5 in RNase-free water). The thermocycling reaction was initiated with (1) the activation of Taq DNA polymerase at 95°C for 3 min, followed by (2) 45 cycles of a 10 sec-denaturation step at 95°C, a 30 sec-annealing step at the optimal primer temperature of annealing (55°C) and a 30 sec-elongation step at 72°C. The fluorescence was measured after the extension step by the iQ5 Multicolor Real-Time PCR Detection System (BioRad). After the 45 cycles of amplification, a melting curve protocol was performed with 1 min-heating at 55°C, followed by 80 steps of 10-sec, with a 0.5°C increment at each step. All reactions were performed in duplicate.

Cycle threshold values were established automatically by the iQ5 Optical System Software (BioRad) on the exponential phase and baseline was set to the same fluorescence value for every run. The Pfaffl method was used to quantify the relative gene expression of each sample, considering the amplification efficiencies for each target gene. Amplification efficiency was determined using the formula E=10(-1/S)-1, where S is the slope of the standard curve. Data for each sample was normalized to the endogenous reference gene (Hprt).

Serum corticosterone assay. Serum corticosterone levels were measured from 3-4-month-old mice under basal conditions and after the tube test. After tail vein blood collection, blood samples were kept at room temperature for 30 min and centrifuged for 10 min at 800g. Serum samples were collected from the supernatant and stored at –80°C. Serum immunoreactive corticosterone levels were quantified using a commercial enzyme-linked immunosorbent assay (ELISA) kit, and according to the manufacturer's recommendations (Abnova). Serum samples were diluted 1:100. Standards (0–100 ng/mL) and samples were measured in duplicate using a SpectraMax Gemini EM fluorimeter (Molecular Devices). Data was analyzed using a linear curve fit in Prism (Graphpad).

Data and resource availability. The RNAseq raw data, processed files, other data or resources are available from the corresponding author upon reasonable request.

Supplementary Table 1 | Transcripts with altered expression in the mPFC of ELS mice

Gene	Locus	Fold change (log_2)	q Value	FPKM Control	FPKM ELS
Ablim3	chr18:61799392-61911861	0,66	7,91E-03	5,849	9,246
Agxt2l1	chr3:130617447-130637634	0,78	7,91E-03	5,031	8,622
Aspa	chr11:73304148-73326830	-0,96	7,91E-03	6,588	3,375
Atp10a	chr7:58658134-58830033	0,79	7,91E-03	1,757	3,03
Atp5e	chr2:174461074-174464101	-0,94	7,91E-03	365,278	190,603
Bcas1	chr2:170344786-170427845	-0,82	7,91E-03	11,036	6,257
Bub1b	chr2:118598210-118641592	-1,89	7,91E-03	1,019	0,275
Cacng3	chr7:122671743-122769394	0,67	7,91E-03	30,71	48,787
Car4	chr11:84957753-84966054	1,14	7,91E-03	9,635	21,187
Chrna5	chr9:54980879-55026559	-1,25	7,91E-03	2,134	0,894
Cntnap4	chr8:112569516-112886859	-0,64	7,91E-03	22,178	14,258
Cplx3	chr9:57599991-57606281	-0,99	7,91E-03	3,439	1,732
Cpne5	chr17:29156483-29240931	0,65	7,91E-03	12,685	19,966
Cpne9	chr6:113282306-113305616	0,8	7,91E-03	5,864	10,238
Ctgf	chr10:24595441-24598682	-1,2	7,91E-03	16,366	7,103
Dct	chr14:118012789-118052246	-3,43	7,91E-03	1,129	0,105
Drd1a	chr13:53861092-54055658	-0,78	7,91E-03	9,242	5,386
Drd5	chr5:38319508-38322310	-0,98	7,91E-03	2,644	1,343
Eomes	chr9:118478188-118486132	2,85	7,91E-03	0,058	0,42
Ermn	chr2:58045114-58052752	-0,79	7,91E-03	9,839	5,671
Exph5	chr9:53301669-53381158	0,71	7,91E-03	5,096	8,323
Fabp7	chr10:57784922-57788450	-0,93	7,91E-03	55,952	29,309
Fmo2	chr1:162874339-162898712	2,07	7,91E-03	0,382	1,606
Fmod	chr1:134037514-134048277	1,53	7,91E-03	1,153	3,339
Fn1	chr1:71585472-71653234	0,92	7,91E-03	2,965	5,605
Gjb1	chrX:101377336-101385914	-1,3	7,91E-03	3,628	1,472
Glra2	chrX:165128984-165327704	-0,73	7,91E-03	20,431	12,282
Gm10635	chr9:79436313-79519302	-1,71	7,91E-03	1,899	0,579
Gpr88	chr3:116249558-116253595	0,69	7,91E-03	10,175	16,384
Grm2	chr9:106643106-106656140	0,66	7,91E-03	11,009	17,342
Hcn4	chr9:58823511-58860955	0,97	7,91E-03	0,595	1,165
Hif3a	chr7:17030992-17062427	1,32	7,91E-03	0,187	0,467
Hist1h4h	chr13:23531043-23531521	-0,9	7,91E-03	148,06	79,215
Hrh3	chr2:180099436-180106312	0,66	7,91E-03	26,782	42,262
Hs3st4	chr7:123983180-124398989	-0,87	7,91E-03	24,528	13,445
Ifi27l1	chr12:103434188-103440245	-0,73	7,91E-03	28,005	16,904

Igf2	chr7:142650767-142670356	0,84	7,91E-03	2,882	5,155
Krt80	chr15:101348444-101405828	-1,48	7,91E-03	1,972	0,708
Lmln,Rpl35a	chr16:33056443-33127666	-0,73	7,91E-03	768,77	462,878
Lsm4	chr8:70673230-70678752	-0,79	7,91E-03	22,449	12,965
Mag	chr7:30899104-30914961	-0,7	7,91E-03	24,976	15,413
Mobp	chr9:120149688-120183874	-0,71	7,91E-03	60,372	36,821
Mp68/2010107E04Rik	chr12:111959264-111967062	-0,75	7,91E-03	487,479	289,063
Mrpl33	chr5:31613950-31622644	-0,7	7,91E-03	87,842	53,976
Myh11	chr16:14194526-14291408	0,97	7,91E-03	0,693	1,353
Ndn	chr7:62348276-62349927	-0,85	7,91E-03	190,022	105,237
Ndnf	chr6:65671581-65712439	-0,96	7,91E-03	8,764	4,517
Neurod1	chr2:79452584-79456891	0,91	7,91E-03	3,305	6,202
Nfe2l3	chr6:51432669-51459295	-1,35	7,91E-03	3,471	1,361
Nxph3	chr11:95509845-95514565	-1,17	7,91E-03	28,806	12,828
Olfm3	chr3:114863252-115125256	-0,66	7,91E-03	18,125	11,451
Opalin	chr19:41059845-41077113	-1,19	7,91E-03	14,859	6,508
Padi6	chr4:140727354-140742643	-1,67	7,91E-03	1,77	0,556
Pappa2	chr1:158711730-158960457	-1,86	7,91E-03	0,423	0,117
Paqr5	chr9:61953737-62026839	1,37	7,91E-03	0,449	1,159
Plch2	chr4:154983114-155043451	0,63	7,91E-03	9,005	13,889
Plp1	chrX:136820218-136838572	-0,74	7,91E-03	217,678	130,746
Plxnd1	chr6:115954810-115995005	0,72	7,91E-03	5,264	8,665
Prdm8	chr5:98167290-98187448	0,82	7,91E-03	4,65	8,217
Prss35	chr9:86743291-86758425	-1,25	7,91E-03	4,451	1,869
Ptgds	chr2:25466529-25470044	0,94	7,91E-03	276,573	529,045
Qdpr	chr5:45432126-45450229	-1,01	7,91E-03	42,414	21,106
Rasgrf2	chr13:91879788-91988044	0,68	7,91E-03	24,324	39,069
Rmrp	chr4:43492784-43493059	-0,92	7,91E-03	24688,433	13040,133
Rn45s	chr17:39842996-39848829	-0,93	7,91E-03	70,668	37,165
Rpl22l1	chr3:28805483-28807578	-0,93	7,91E-03	180,635	94,983
Rpl34,Rpl34-ps1	chr3:130726830-130730329	-0,71	7,91E-03	562,032	343,181
Rpl36al	chr12:69182733-69184067	-0,62	7,91E-03	225,654	146,367
Rpl37a	chr1:72711239-72718757	-0,79	7,91E-03	727,943	421,465
Rpl39	chrX:37082519-37085184	-0,87	7,91E-03	518,836	283,887
Rpl9	chr5:65388363-65391431	-0,78	7,91E-03	888,273	518,374
Rplp2	chr7:141447649-141451342	-0,69	7,91E-03	495,059	306,186
Rps12	chr10:23785182-23787209	-0,63	7,91E-03	665,705	431,592
Rps14	chr18:60774595-60778546	-0,92	7,91E-03	823,038	434,561
Rps18	chr17:33951998-33955641	-0,61	7,91E-03	433,423	284,077

Rps27a	chr11:29545841-29578352	-0,83	7,91E-03	247,188	138,603
Rps3	chr7:99477896-99483709	-0,76	7,91E-03	232,061	137,088
Rps7	chr12:28627278-28635953	-0,61	7,91E-03	434,912	284,177
Scarna13	chr12:105030616-105032279	-1,07	7,91E-03	1513,724	721,69
Scarna2	chr3:108553693-108554879	-1,1	7,91E-03	107,436	50,039
Scn7a	chr2:66673265-66785024	0,66	7,91E-03	2,607	4,119
Sema5a	chr15:32244812-32696341	-0,72	7,91E-03	9,627	5,854
Serping1	chr2:84765359-84775429	1,39	7,91E-03	0,836	2,193
Sla	chr15:66670769-66850720	-0,8	7,91E-03	10,883	6,234
Slc13a4	chr6:35267952-35308126	1,15	7,91E-03	1,815	4,018
Slc22a6	chr19:8617995-8628299	1,11	7,91E-03	0,811	1,747
Slc22a8	chr19:8591202-8611973	0,91	7,91E-03	1,802	3,393
Slc30a3	chr5:31086105-31093527	0,83	7,91E-03	22,601	40,206
Spag16	chr1:69826969-70725132	-2,31	7,91E-03	2,481	0,502
Stra6	chr9:58129087-58154656	1,12	7,91E-03	0,8	1,742
Sulf1	chr1:12691711-12861191	-0,95	7,91E-03	9,35	4,833
Sult1a1	chr7:126672800-126676422	1,1	7,91E-03	3,083	6,618
Tac1	chr6:7554763-7563092	-0,84	7,91E-03	22,523	12,579
Tmem88b	chr4:155781476-155785874	-0,76	7,91E-03	6,18	3,64
Trf	chr9:103208875-103230286	-0,84	7,91E-03	64,266	35,817
Uqcrq	chr11:53428733-53430831	-1,28	7,91E-03	104,87	43,228
Vstm2l	chr2:157914652-157944719	0,9	7,91E-03	26,294	48,934
Vwf	chr6:125552947-125686679	0,93	7,91E-03	1,363	2,595
Wfs1	chr5:36966103-36988982	0,76	7,91E-03	12,617	21,357
Xdh	chr17:73883907-73950182	1,14	7,91E-03	0,848	1,864
Zfhx4	chr3:5177827-5415855	0,96	7,91E-03	0,638	1,243
Bgn	chrX:73483634-73495936	1,02	1,43E-02	1,243	2,516
Calca	chr7:114631477-114636785	-1,66	1,43E-02	2,284	0,723
Enpp6	chr8:46986922-47096753	-0,99	1,43E-02	1,207	0,607
Hint2	chr4:43654226-43656445	-0,82	1,43E-02	35,436	20,064
Hkdc1	chr10:62383070-62422457	0,72	1,43E-02	4,161	6,853
Lamp5	chr2:136052312-136069983	0,66	1,43E-02	35,626	56,467
Serpinb1a	chr13:32842091-32851185	-1,2	1,43E-02	2,003	0,87
Stard8	chrX:99003269-99074728	0,65	1,43E-02	6,169	9,655
Tbc1d4	chr14:101442359-101609191	0,79	1,43E-02	1,171	2,02
Trim21	chr7:102557921-102565469	-1,03	1,43E-02	1,455	0,712
Zfpm1	chr8:122281889-122342680	0,74	1,43E-02	4,681	7,802
Cnp	chr11:100574938-100581739	-0,62	1,96E-02	57,316	37,4
Cux2	chr5:121857746-122050200	0,85	1,96E-02	12,757	22,941

Hist1h4m	chr13:21722078-22041352	-1,27	1,96E-02	150,544	62,437
Mgat5b	chr11:116918862-116986944	0,62	1,96E-02	13,284	20,371
Rps21	chr2:180257378-180273465	-0,68	1,96E-02	1304,22	813,651
Smad3	chr9:63646605-63769348	0,61	1,96E-02	14,5	22,093
Thbs4	chr13:92751585-92794818	-1,87	1,96E-02	0,524	0,143
Tmem215	chr4:40472179-40477125	1,1	1,96E-02	0,716	1,531
Vwa5b1	chr4:138566705-138623992	1,15	1,96E-02	0,319	0,709
E130012A19Rik	chr11:97627306-97629856	0,66	2,52E-02	14,092	22,265
Net1	chr13:3882560-3918220	0,68	2,52E-02	7,551	12,065
Pdlim2	chr14:70164180-70177672	-0,87	2,52E-02	4,351	2,381
Rplp1	chr9:61913282-61914510	-0,62	2,52E-02	1642,8	1068,937
Arhgap31	chr16:38598123-38713035	0,64	2,86E-02	3,019	4,707
Atp5l	chr9:44913247-44920742	-0,6	2,86E-02	413,825	273,304
Cpne4	chr9:104566722-105034632	0,58	2,86E-02	17,195	25,756
Dohh	chr10:81384427-81388352	-0,59	2,86E-02	77,334	51,362
Gpr153	chr4:152274129-152285339	0,71	2,86E-02	4,606	7,514
Igfn1	chr1:135953499-136006342	0,85	2,86E-02	0,524	0,944
Lgr5	chr10:115450313-115587780	1,13	2,86E-02	0,287	0,63
Nr4a3	chr4:48045082-48086449	-0,63	2,86E-02	9,416	6,094
Per2	chr1:91415981-91459328	0,6	2,86E-02	4,926	7,457
Plagl1	chr10:13090692-13131695	0,74	2,86E-02	2,158	3,608
Rps23	chr13:90923121-90924732	-0,57	2,86E-02	649,966	436,464
Sdhaf4/1110058L19Rik	chr1:23995938-24005640	-0,7	2,86E-02	35,345	21,815
Sema5b	chr16:35541080-35664724	-0,72	2,86E-02	3,814	2,32
Slc2a1	chr4:119108738-119138549	0,55	2,86E-02	24,753	36,347
Abcc4	chr14:118482691-118706219	0,75	3,28E-02	1,389	2,33
Aif1l	chr2:31950289-31973563	-0,8	3,28E-02	2,862	1,644
Ddit4l	chr3:137623671-137628332	0,73	3,28E-02	2,742	4,545
Fmc1/1110001J03Rik	chr6:38534823-38541096	-0,77	3,28E-02	54,59	31,966
Foxp2	chr6:14901180-15442176	-0,64	3,28E-02	4,535	2,913
Abi3bp	chr16:56477845-56690205	0,8	3,65E-02	0,923	1,606
Mog	chr17:37001162-37023398	-0,85	3,65E-02	10,415	5,779
Palmd	chr3:116917681-116984406	0,65	3,65E-02	5,794	9,113
Ppp1r1b	chr11:98348737-98358283	-0,66	3,65E-02	23,872	15,056
Rasd2	chr8:75213943-75224113	0,59	3,65E-02	10,377	15,657
Rps19	chr7:24884574-24889941	-0,62	3,65E-02	349,067	227,391
Thsd4/9230112J17Rik	chr9:59966930-60545882	0,7	3,65E-02	1,726	2,8
Car10	chr11:93097962-93601751	0,59	3,91E-02	32,028	48,266
Fancd2	chr6:113531681-113600715	-0,96	3,91E-02	4,739	2,444

Kirrel2	chr7:30447765-30457515	0,82	3,91E-02	1,368	2,42
Lpar1	chr4:58435154-58553491	-0,69	3,91E-02	7,277	4,524
Mal	chr2:127633225-127656695	-0,56	3,91E-02	43,154	29,259
Ndufb6	chr4:40270662-40279368	-0,59	3,91E-02	151,777	100,921
Nme2	chr11:93949814-93956007	-0,6	3,91E-02	135,474	89,261
Rpph1	chr14:50807446-50807771	-0,9	3,91E-02	63576,7	33972,067
Scube1	chr15:83602529-83725039	0,59	3,91E-02	7,443	11,232
Stmn4	chr14:66344234-66361896	-0,66	3,91E-02	123,903	78,242
Syt17	chr7:118380714-118443818	0,6	3,91E-02	33,082	50,063
Ccbe1	chr18:66056855-66291838	0,65	4,25E-02	1,521	2,389
Crym	chr7:120186383-120201988	-0,56	4,25E-02	169,889	114,93
D8Ertd82e	chr8:36094827-36147787	0,59	4,25E-02	4,948	7,434
Mctp2	chr7:72077829-72306595	-1,05	4,25E-02	0,597	0,289
Pdzrn3	chr6:101149606-101377897	0,58	4,25E-02	9,337	13,921
Prelp	chr1:133910303-133921401	0,81	4,25E-02	1,207	2,116
Insc	chr7:114745563-114850380	-1,18	4,63E-02	1,111	0,491
Npy1r	chr8:66696939-66706918	0,62	4,63E-02	9,129	13,984
Rpl36	chr17:56613394-56614246	-0,57	4,63E-02	532,861	359,008
Tmem108	chr9:103482935-103761908	0,71	4,63E-02	2,58	4,232
Arhgap25	chr6:87458468-87533316	-0,67	4,88E-02	8,118	5,093
Cited4	chr4:120666562-120667820	0,79	4,88E-02	3,558	6,17
Cwh43	chr5:73406072-73453425	-0,94	4,88E-02	1,546	0,806
Fdps	chr3:89093587-89101967	-0,56	4,88E-02	92,848	62,772
Gata2	chr6:88198663-88207032	1,41	4,88E-02	0,24	0,637
Rpl13	chr8:123102349-123105242	-0,55	4,88E-02	551,427	377,919
Rpl26	chr11:68901534-68906996	-0,58	4,88E-02	915,055	611,04
Tubb2b	chr13:34127007-34130354	-0,58	4,88E-02	36,127	24,248

FPKM, fragments per kilobase of transcript per million fragments mapped.

Supplementary Table 2 | Top 60 GOs for Biological Process

Gene Ontology	# Transcripts	FDR	FDR < 0,05
cellular process (GO:0009987)	134	3,55E-04	*
developmental process (GO:0032502)	68	3,61E-04	*
positive regulation of biological process (GO:0048518)	67	3,41E-03	*
anatomical structure development (GO:0048856)	64	7,45E-04	*
multicellular organism development (GO:0007275)	61	1,05E-03	*
positive regulation of cellular process (GO:0048522)	61	7,06E-03	*
system development (GO:0048731)	58	2,83E-04	*
cellular developmental process (GO:0048869)	54	1,70E-04	*
cell differentiation (GO:0030154)	53	2,13E-04	*
regulation of biological quality (GO:0065008)	51	4,88E-04	*
biosynthetic process (GO:0009058)	50	3,30E-03	*
organic substance biosynthetic process (GO:1901576)	49	3,96E-03	*
cellular biosynthetic process (GO:0044249)	48	4,95E-03	*
regulation of multicellular organismal process (GO:0051239)	43	1,95E-03	*
regulation of cell communication (GO:0010646)	42	7,21E-03	*
regulation of signaling (GO:0023051)	42	7,33E-03	*
cellular nitrogen compound biosynthetic process (GO:0044271)	41	2,32E-03	*
cellular macromolecule biosynthetic process (GO:0034645)	39	1,68E-02	*
nervous system development (GO:0007399)	38	3,62E-04	*
regulation of developmental process (GO:0050793)	36	1,32E-02	*
positive regulation of multicellular organismal process (GO:0051240)	34	1,31E-04	*
neurogenesis (GO:0022008)	32	3,50E-04	*
generation of neurons (GO:0048699)	31	3,34E-04	*
regulation of cell differentiation (GO:0045595)	30	4,98E-03	*
cell development (GO:0048468)	28	6,94E-03	*
positive regulation of developmental process (GO:0051094)	27	2,10E-03	*
organonitrogen compound biosynthetic process (GO:1901566)	26	1,72E-04	*
regulation of cell development (GO:0060284)	24	3,34E-04	*
positive regulation of cell differentiation (GO:0045597)	24	4,46E-04	*

positive regulation of cell development (GO:0010720)	20	6,40E-05	*
translation (GO:0006412)	19	5,85E-08	*
peptide biosynthetic process (GO:0043043)	19	7,98E-08	*
amide biosynthetic process (GO:0043604)	19	1,11E-06	*
peptide metabolic process (GO:0006518)	19	4,30E-06	*
cellular amide metabolic process (GO:0043603)	19	1,30E-04	*
regulation of neurogenesis (GO:0050767)	19	6,94E-03	*
**behavior (GO:0007610)**	**18**	**1,25E-03**	*****
positive regulation of neurogenesis (GO:0050769)	17	3,74E-04	*
positive regulation of nervous system development (GO:0051962)	17	1,36E-03	*
central nervous system development (GO:0007417)	17	1,67E-02	*
regulation of growth (GO:0040008)	16	1,38E-02	*
positive regulation of neuron differentiation (GO:0045666)	14	2,13E-03	*
regulation of cell morphogenesis (GO:0022604)	14	5,46E-03	*
response to toxic substance (GO:0009636)	12	2,38E-03	*
positive regulation of neuron projection development (GO:0010976)	12	3,36E-03	*
anterograde trans-synaptic signaling (GO:0098916)	11	1,70E-02	*
chemical synaptic transmission (GO:0007268)	11	1,73E-02	*
learning or memory (GO:0007611)	10	1,17E-02	*
cytoplasmic translation (GO:0002181)	8	1,01E-05	*
regulation of cAMP metabolic process (GO:0030814)	8	3,39E-03	*
regulation of cyclic nucleotide biosynthetic process (GO:0030802)	8	3,48E-03	*
regulation of purine nucleotide biosynthetic process (GO:1900371)	8	5,40E-03	*
regulation of nucleotide biosynthetic process (GO:0030808)	8	5,54E-03	*
regulation of cyclic nucleotide metabolic process (GO:0030799)	8	7,97E-03	*
learning (GO:0007612)	8	1,04E-02	*
feeding behavior (GO:0007631)	7	7,70E-03	*
regulation of cAMP biosynthetic process (GO:0030817)	7	9,24E-03	*
positive regulation of cyclic nucleotide biosynthetic process (GO:0030804)	6	1,52E-02	*
positive regulation of adenylate cyclase activity (GO:0045762)	4	1,76E-02	*
sensory perception of chemical stimulus (GO:0007606)	0	1,51E-02	*

Supplementary Table 3 | Top 60 GOs for Cellular Component

Gene Ontology	# Transcripts	FDR	FDR < 0,05
cellular_component (GO:0005575)	166	6,11E-03	*
cell (GO:0005623)	145	6,36E-06	*
cell part (GO:0044464)	145	6,94E-06	*
organelle (GO:0043226)	121	5,27E-04	*
intracellular (GO:0005622)	121	1,30E-02	*
intracellular part (GO:0044424)	119	2,37E-02	*
cytoplasm (GO:0005737)	113	7,85E-06	*
membrane-bounded organelle (GO:0043227)	112	3,02E-03	*
intracellular organelle (GO:0043229)	106	4,62E-02	*
cytoplasmic part (GO:0044444)	94	6,49E-06	*
membrane (GO:0016020)	90	1,04E-01	ns
cell periphery (GO:0071944)	65	8,78E-05	*
extracellular region (GO:0005576)	63	2,00E-05	*
extracellular region part (GO:0044421)	58	1,79E-05	*
plasma membrane (GO:0005886)	58	5,22E-03	*
extracellular space (GO:0005615)	54	1,11E-04	*
vesicle (GO:0031982)	54	2,34E-04	*
cytosol (GO:0005829)	43	1,25E-03	*
extracellular exosome (GO:0070062)	41	2,05E-04	*
extracellular vesicle (GO:1903561)	41	2,23E-04	*
extracellular organelle (GO:0043230)	41	2,25E-04	*
cell projection (GO:0042995)	36	4,57E-04	*
plasma membrane bounded cell projection (GO:0120025)	32	3,55E-03	*
neuron part (GO:0097458)	27	6,19E-03	*
cell junction (GO:0030054)	24	1,27E-03	*
neuron projection (GO:0043005)	23	1,01E-02	*
ribonucleoprotein complex (GO:1990904)	21	7,78E-05	*
intracellular ribonucleoprotein complex (GO:0030529)	21	7,94E-05	*
ribosomal subunit (GO:0044391)	19	1,30E-12	*

ribosome (GO:0005840)	19	2,22E-11	*
**synapse (GO:0045202)**	**19**	**5,35E-03**	*****
cytosolic ribosome (GO:0022626)	18	1,59E-14	*
cytosolic part (GO:0044445)	18	6,37E-10	*
extracellular matrix (GO:0031012)	18	1,44E-05	*
plasma membrane region (GO:0098590)	18	1,63E-02	*
somatodendritic compartment (GO:0036477)	17	2,59E-02	*
synapse part (GO:0044456)	15	2,65E-02	*
large ribosomal subunit (GO:0015934)	11	1,10E-06	*
proteinaceous extracellular matrix (GO:0005578)	11	5,14E-03	*
axon (GO:0030424)	11	7,68E-02	ns
cytosolic large ribosomal subunit (GO:0022625)	10	6,83E-08	*
cytosolic small ribosomal subunit (GO:0022627)	9	5,94E-08	*
small ribosomal subunit (GO:0015935)	9	1,96E-06	*
myelin sheath (GO:0043209)	9	2,80E-03	*
focal adhesion (GO:0005925)	9	7,12E-02	ns
cell-substrate adherens junction (GO:0005924)	9	7,64E-02	ns
cell-substrate junction (GO:0030055)	9	8,17E-02	ns
adherens junction (GO:0005912)	9	3,27E-01	ns
anchoring junction (GO:0070161)	9	3,71E-01	ns
presynapse (GO:0098793)	8	2,79E-01	ns
cell cortex (GO:0005938)	6	3,63E-01	ns
plasma membrane raft (GO:0044853)	4	3,78E-01	ns
inner mitochondrial membrane protein complex (GO:0098800)	4	4,02E-01	ns
endoplasmic reticulum-Golgi intermediate compartment (GO:0005793)	3	3,54E-01	ns
sarcoplasmic reticulum (GO:0016529)	3	4,02E-01	ns
myelin sheath adaxonal region (GO:0035749)	2	6,39E-02	ns
paranode region of axon (GO:0033270)	2	2,87E-01	ns
mitochondrial proton-transporting ATP synthase complex (GO:0005753)	2	3,40E-01	ns
proton-transporting ATP synthase complex (GO:0045259)	2	3,74E-01	ns
Unclassified (UNCLASSIFIED)	1	5,94E-03	*

Supplementary Table 4 | Top 60 GOs for Molecular Function

Gene Ontology	# Transcripts	FDR	FDR < 0,05
molecular_function (GO:0003674)	164	9,21E-02	ns
binding (GO:0005488)	129	1,43E-04	*
protein binding (GO:0005515)	97	6,63E-04	*
ion binding (GO:0043167)	57	4,98E-01	ns
structural molecule activity (GO:0005198)	26	8,46E-10	*
identical protein binding (GO:0042802)	23	9,94E-01	ns
protein dimerization activity (GO:0046983)	22	1,46E-01	ns
RNA binding (GO:0003723)	22	6,33E-01	ns
molecular function regulator (GO:0098772)	22	8,79E-01	ns
structural constituent of ribosome (GO:0003735)	19	2,14E-13	*
transporter activity (GO:0005215)	18	8,26E-01	ns
**receptor activity (GO:0004872)**	**16**	**1,02E+00**	**ns**
protein homodimerization activity (GO:0042803)	15	6,11E-01	ns
transcription regulatory region sequence-specific DNA binding (GO:0000976)	13	9,68E-01	ns
RNA polymerase II regulatory region sequence-specific DNA binding (GO:0000977)	12	8,62E-01	ns
RNA polymerase II regulatory region DNA binding (GO:0001012)	12	8,94E-01	ns
glycosaminoglycan binding (GO:0005539)	8	8,87E-02	ns
sulfur compound binding (GO:1901681)	8	1,49E-01	ns
heparin binding (GO:0008201)	6	2,26E-01	ns
collagen binding (GO:0005518)	5	9,22E-02	ns
activating transcription factor binding (GO:0033613)	5	1,00E-01	ns
rRNA binding (GO:0019843)	5	1,06E-01	ns
neurotransmitter receptor activity (GO:0030594)	5	3,16E-01	ns
RNA polymerase II transcription factor binding (GO:0001085)	5	6,04E-01	ns
RNA polymerase II activating transcription factor binding (GO:0001102)	4	1,09E-01	ns
structural constituent of myelin sheath (GO:0019911)	3	9,88E-02	ns
Unclassified (UNCLASSIFIED)	3	1,02E-01	ns
mRNA 5'-UTR binding (GO:0048027)	3	2,06E-01	ns
G-protein alpha-subunit binding (GO:0001965)	3	4,95E-01	ns

fatty acid binding (GO:0005504)	3	7,16E-01	ns
NADP binding (GO:0050661)	3	7,35E-01	ns
D3 dopamine receptor binding (GO:0031750)	2	2,03E-01	ns
dopamine neurotransmitter receptor activity, coupled via Gs (GO:0001588)	2	2,15E-01	ns
small ribosomal subunit rRNA binding (GO:0070181)	2	5,11E-01	ns
neuropilin binding (GO:0038191)	2	7,88E-01	ns
dopamine neurotransmitter receptor activity (GO:0004952)	2	8,15E-01	ns
hydrogen-exporting ATPase activity, phosphorylative mechanism (GO:0008553)	2	8,42E-01	ns
proton-transporting ATP synthase activity, rotational mechanism (GO:0046933)	2	8,43E-01	ns
dopamine binding (GO:0035240)	2	9,49E-01	ns
oxidoreductase activity, acting on the CH-NH group of donors, NAD or NADP as acceptor (GO:0016646)	2	1,00E+00	ns
growth factor receptor binding (GO:0070851)	1	1,00E+00	ns
transferase activity, transferring hexosyl groups (GO:0016758)	1	1,00E+00	ns
oxidoreductase activity, acting on CH-OH group of donors (GO:0016614)	1	1,00E+00	ns
phosphatidylinositol phosphate binding (GO:1901981)	1	1,00E+00	ns
UDP-glycosyltransferase activity (GO:0008194)	1	1,00E+00	ns
S-adenosylmethionine-dependent methyltransferase activity (GO:0008757)	1	1,00E+00	ns
carboxylic acid transmembrane transporter activity (GO:0046943)	1	1,01E+00	ns
catalytic activity, acting on DNA (GO:0140097)	1	1,01E+00	ns
organic acid transmembrane transporter activity (GO:0005342)	1	1,01E+00	ns
serine-type endopeptidase activity (GO:0004252)	1	1,01E+00	ns
transferase activity, transferring one-carbon groups (GO:0016741)	1	1,01E+00	ns
protein tyrosine kinase activity (GO:0004713)	1	1,01E+00	ns
nuclease activity (GO:0004518)	1	1,01E+00	ns
microtubule binding (GO:0008017)	1	1,02E+00	ns
serine hydrolase activity (GO:0017171)	1	1,02E+00	ns
cytokine activity (GO:0005125)	1	1,02E+00	ns
protein phosphatase binding (GO:0019903)	1	1,02E+00	ns
magnesium ion binding (GO:0000287)	1	1,02E+00	ns
phosphatidylinositol binding (GO:0035091)	1	1,02E+00	ns
metallopeptidase activity (GO:0008237)	1	1,02E+00	ns

Supplementary Table 5 | Altered Transcripts from Gene Ontology Behavior

Gene	Fold Change (log_2)	q-value
Chrna5	-1,25	0,00790727
Drd1a	-0,78	0,00790727
Drd5	-0,98	0,00790727
Gpr88	0,69	0,00790727
Hrh3	0,66	0,00790727
Igf2	0,84	0,00790727
Stra6	1,12	0,00790727
Tac1	-0,84	0,00790727
Trf	-0,84	0,00790727
Calca	-1,66	0,014259
Cnp	-0,62	0,0196195
Cux2	0,85	0,0196195
Thbs4	-1,87	0,0196195
Nr4a3	-0,63	0,0286118
Foxp2	-0,64	0,0328226
Ppp1r1b	-0,66	0,0364587
Rasd2	0,59	0,0364587
Npy1r	0,62	0,046266

Supplementary Table 6 | Altered Transcripts from Gene Ontology Synapse

Gene	Fold Change (log_2)	q-value
Chrna5	-1,25	0,00790727
Drd1a	-0,78	0,00790727
Drd5	-0,98	0,00790727
Glra2	-0,73	0,00790727
Grm2	0,66	0,00790727
Bcas1	-0,82	0,00790727
Cntnap4	-0,64	0,00790727
Cplx3	-0,99	0,00790727
Hcn4	0,97	0,00790727
Olfm3	-0,66	0,00790727
Slc30a3	0,83	0,00790727
Calca	-1,66	0,014259
Thbs4	-1,87	0,0196195
Palmd	0,65	0,0364587
Lpar1	-0,69	0,0390921
Pdzrn3	0,58	0,0425446
Npy1r	0,62	0,046266
Tmem108	0,71	0,046266
Rpl26	-0,58	0,0488153

Supplementary Table 7 | Altered Transcripts from Gene Ontology Receptor Activity

Gene	Fold Change (log_2)	q Value
Chrna5	-1,25	0,00790727
Drd1a	-0,78	0,00790727
Drd5	-0,98	0,00790727
Glra2	-0,73	0,00790727
Grm2	0,66	0,00790727
Gpr88	0,69	0,00790727
Hrh3	0,66	0,00790727
Stra6	1,12	0,00790727
Paqr5	1,37	0,00790727
Plxnd1	0,72	0,00790727
Sema5a	-0,72	0,00790727
Nr4a3	-0,63	0,0286118
Gpr153	0,71	0,0286118
Lgr5	1,13	0,0286118
Lpar1	-0,69	0,0390921
Npy1r	0,62	0,046266

Supplementary References:

1. Edfawy M, Guedes JR, Pereira MI, Laranjo M, Carvalho MJ, Gao X, et al. Abnormal mGluR-mediated synaptic plasticity and autism-like behaviours in Gprasp2 mutant mice. Nat Commun. 2019;10(1):1431.
2. Trapnell C, Roberts A, Goff L, Pertea G, Kim D, Kelley DR, et al. Differential gene and transcript expression analysis of RNA-seq experiments with TopHat and Cufflinks. Nature protocols. 2012;7(3):562.
