# Supplementary figures and images for: Social subordination induced by early life adversity rewires inhibitory control of the prefrontal cortex via enhanced Npy1r signaling
Source: Neuropsychopharmacology. 2020 Jun 3;45(9):1438–47. doi: 10.1038/s41386-020-0727-7 (PMC7360628; doi:10.1038/s41386-020-0727-7)

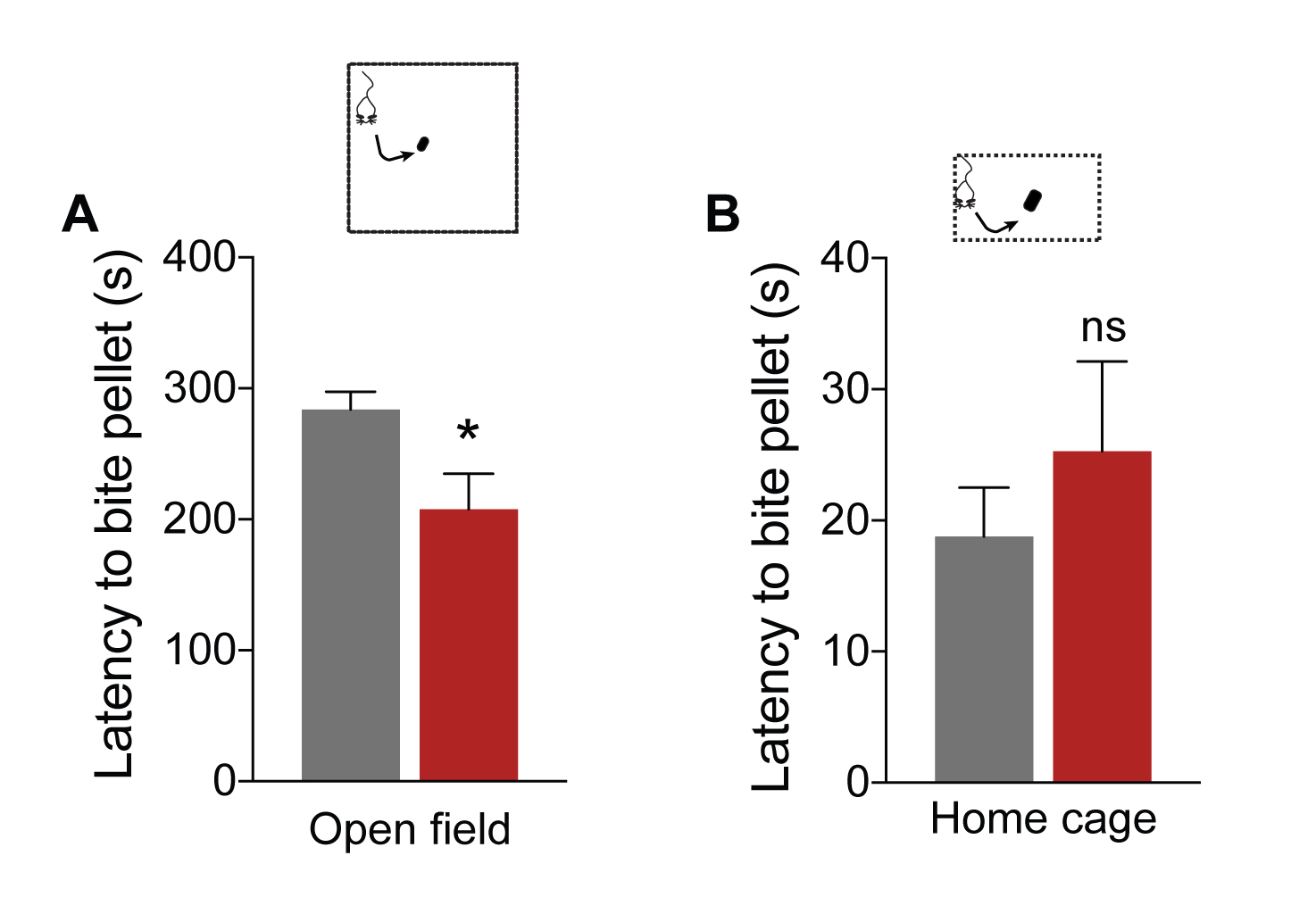

Supplement: Supplementary file 2 — Supplementary Figure 1 [file 41386_2020_727_MOESM2_ESM.jpg]

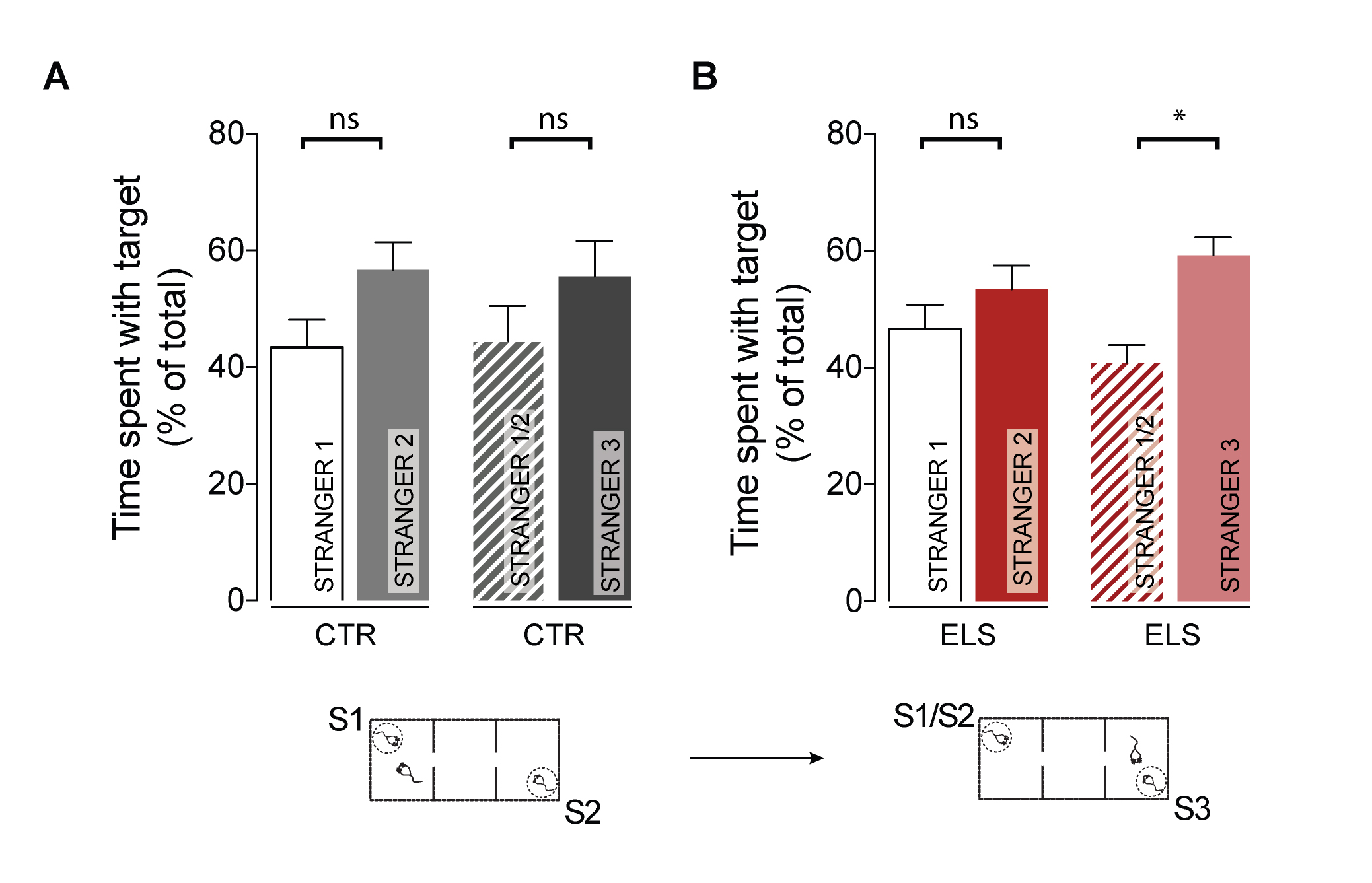

Supplement: Supplementary file 3 — Supplementary Figure 2 [file 41386_2020_727_MOESM3_ESM.jpg]

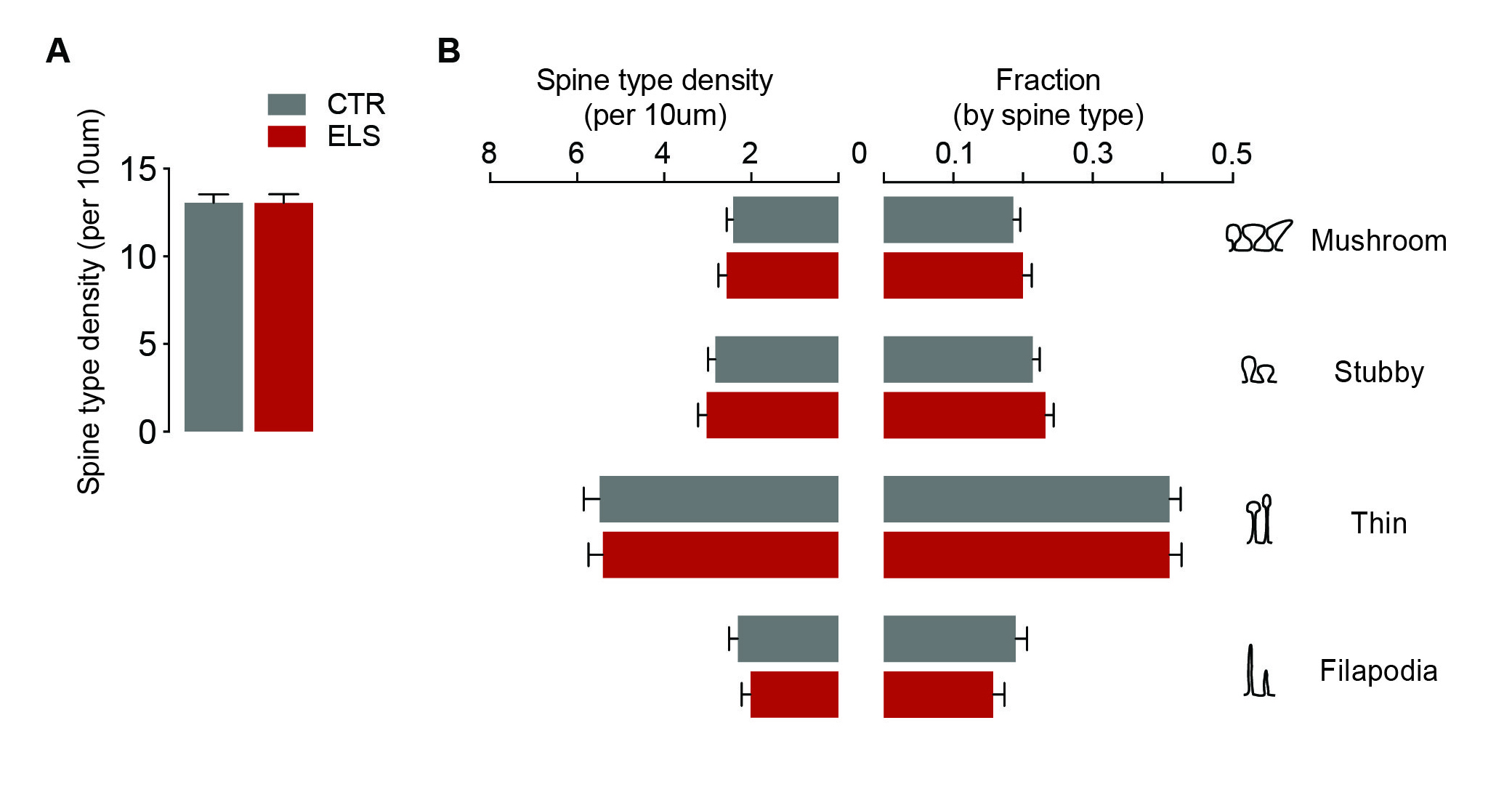

Supplement: Supplementary file 4 — Supplementary Figure 3 [file 41386_2020_727_MOESM4_ESM.jpg]

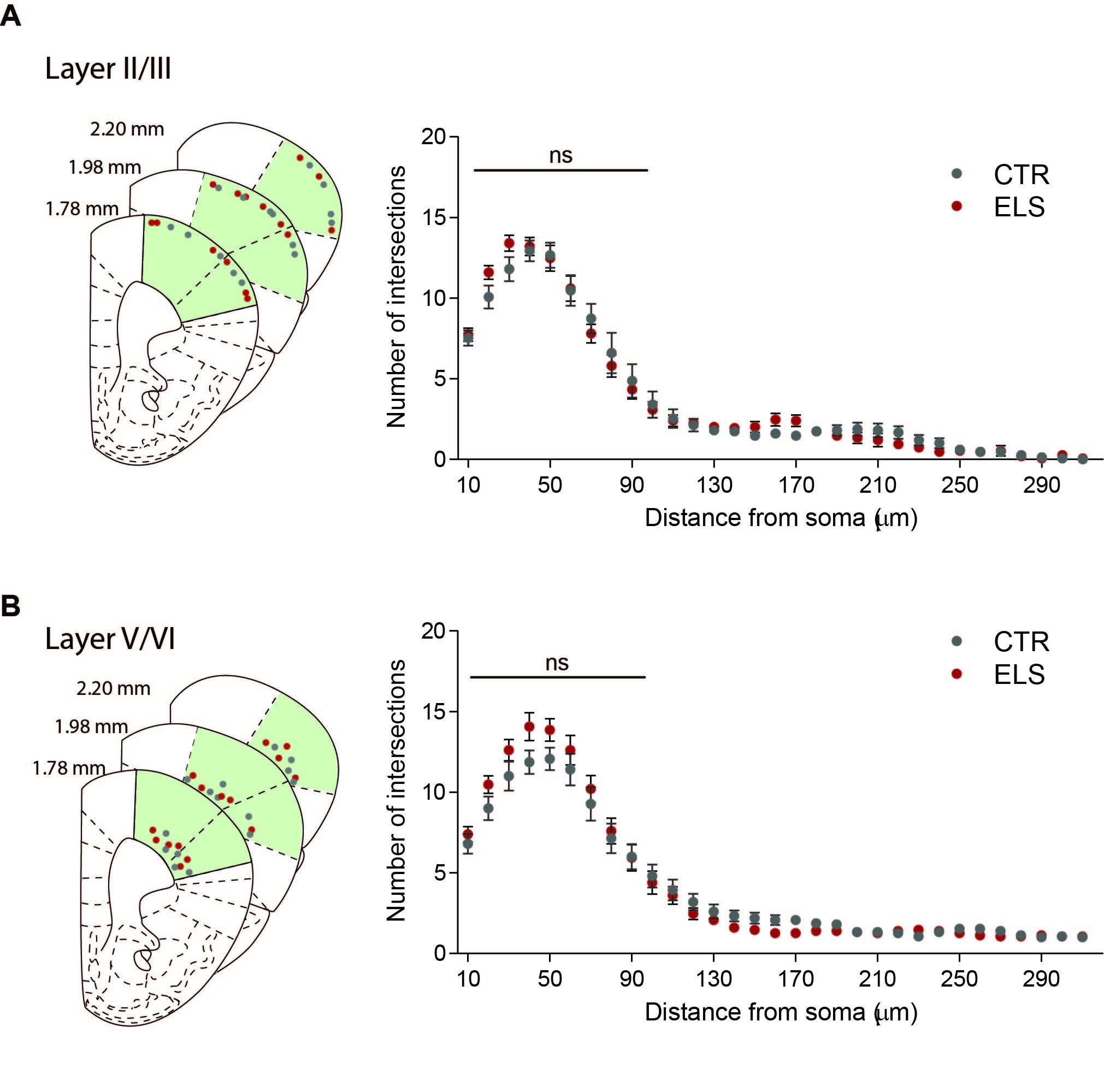

Supplement: Supplementary file 5 — Supplementary Figure 4 [file 41386_2020_727_MOESM5_ESM.jpg]

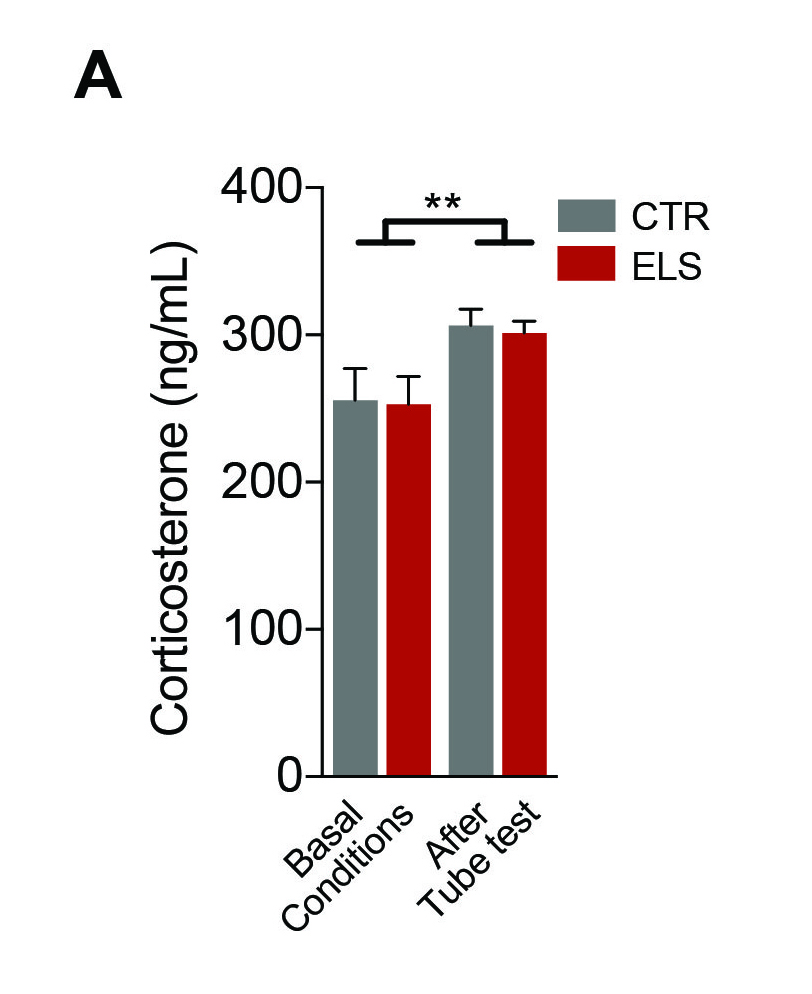

Supplement: Supplementary file 6 — Supplementary Figure 5 [file 41386_2020_727_MOESM6_ESM.jpg]

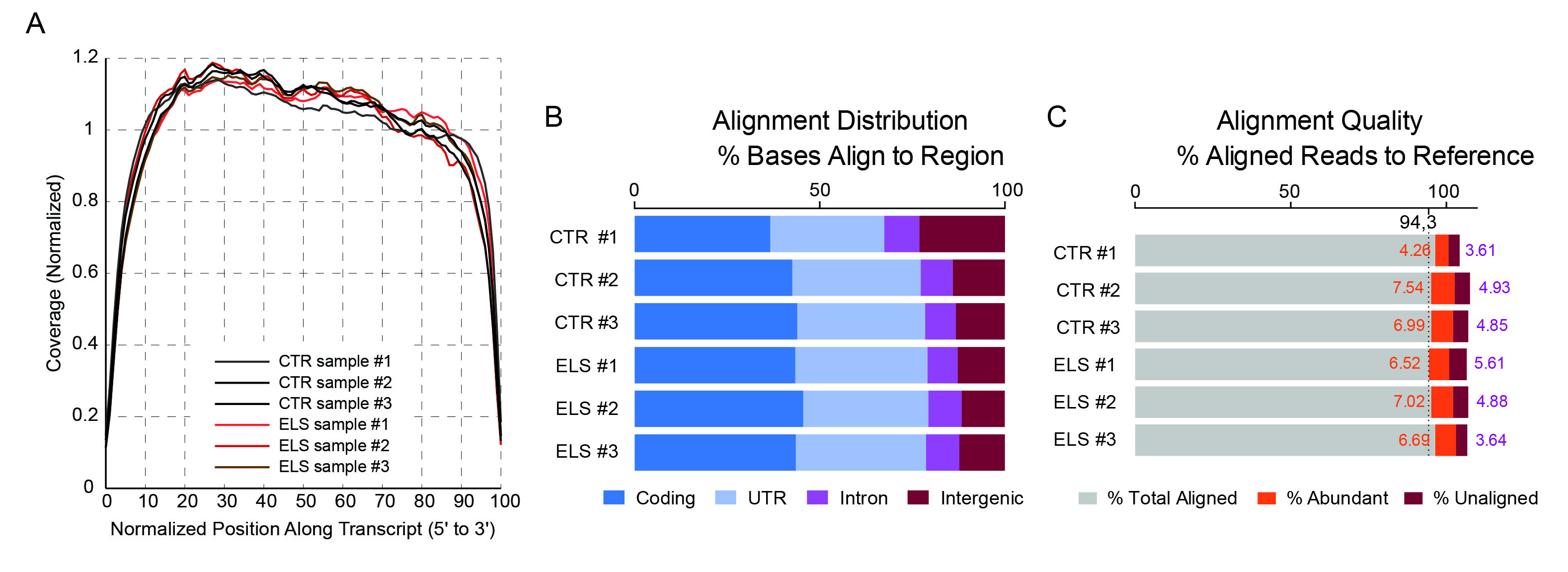

Supplement: Supplementary file 7 — Supplementary Figure 6 [file 41386_2020_727_MOESM7_ESM.jpg]
